# SMG6 regulates DNA damage and cell survival in Hippo pathway kinase LATS2-inactivated malignant mesothelioma

**DOI:** 10.1038/s41420-022-01232-w

**Published:** 2022-11-05

**Authors:** Koya Suzuki, Masaki Tange, Ryota Yamagishi, Hiroyuki Hanada, Satomi Mukai, Tatsuhiro Sato, Takeshi Tanaka, Tomohiro Akashi, Kenji Kadomatsu, Tohru Maeda, Takashi Miida, Ichiro Takeuchi, Hiroshi Murakami, Yoshitaka Sekido, Yuko Murakami-Tonami

**Affiliations:** 1 Department of Clinical Laboratory of Medicine, Juntendo University Graduate School of Medicine, Tokyo, Japan; 2 Research Institute for Diseases of Old Age, Juntendo University Graduate School of Medicine, Tokyo, Japan; 3 Cancer Molecular Genetics Lab, Tokyo University of Technology Graduate School of Bionics, Tokyo, Japan; 4 Advanced Comprehensive Research Organization, Teikyo University, Tokyo, Japan; 5 Department of Pathophysiology, Osaka Metropolitan University Graduate School of Medicine, Osaka, Japan; 6 Center for Advanced Intelligence Project, RIKEN, Tokyo, Japan; 7 Division of Cancer Biology, Aichi Cancer Center Research Institute, Nagoya, Japan; 8 Interprotein Corporation, Sagamihara, Japan; 9 Department of Integrative Cellular Informatics, Nagoya University Graduate School of Medicine, Nagoya, Japan; 10 Department of Biochemistry, Nagoya University Graduate School of Medicine, Nagoya, Japan; 11 Institute for Glyco-core Research (iGCORE), Nagoya University, Nagoya, Japan; 12 College of Pharmacy, Kinjo Gakuin University, Nagoya, Japan; 13 Graduate School of Engineering, Nagoya University, Nagoya, Japan; 14 Department of Biological Sciences, Faculty of Science and Engineering, Chuo University, Tokyo, Japan; 15 Division of Molecular and Cellular Oncology, Nagoya University Graduate School of Medicine, Nagoya, Japan

**Keywords:** Mesothelioma, HIPPO signalling

## Abstract

Many genes responsible for Malignant mesothelioma (MM) have been identified as tumor suppressor genes and it is difficult to target these genes directly at a molecular level. We searched for the gene which showed synthetic lethal phenotype with LATS2, one of the MM causative genes and one of the kinases in the Hippo pathway. Here we showed that knockdown of SMG6 results in synthetic lethality in LATS2-inactivated cells. We found that this synthetic lethality required the nuclear translocation of YAP1 and TAZ. Both are downstream factors of the Hippo pathway. We also demonstrated that this synthetic lethality did not require SMG6 in nonsense-mediated mRNA decay (NMD) but in regulating telomerase reverse transcriptase (TERT) activity. In addition, the RNA-dependent DNA polymerase (RdDP) activity of TERT was required for this synthetic lethal phenotype. We confirmed the inhibitory effects of LATS2 and SMG6 on cell proliferation in vivo. The result suggests an interaction between the Hippo and TERT signaling pathways. We also propose that SMG6 and TERT are novel molecular target candidates for LATS2-inactivated cancers such as MM.

## Introduction

Malignant mesothelioma (MM) is an aggressive tumor and patients with an advanced stage of MM usually show a very poor prognosis. Furthermore, MM is lethally refractory to general treatment for cancer, including surgery and radiation therapy. Currently, chemotherapy regimens improve survival by only a few months [1–3]. The results of the Checkmate 743 trial (Phase III trial) using the immune checkpoint inhibitors nivolumab and ipilimumab reported improved prognosis. Still, they are not effective in all patients [4]. Therefore, the search for new molecular targets has become urgent.

It has been reported that LATS2, NF2, BAP1, SETDB1, and SETD2 tumor suppressor genes were identified as causative genes of MM [5, 6]. Recently, synthetic lethality has attracted attention as an effective strategy against cancers with tumor suppressor gene mutations [7–10]. The genetic concept of synthetic lethality describes an interaction between two or more mutations that are well tolerated individually but lethal when combined. When synthetic lethality is used to search for new molecular targets, one gene is identified as the causative gene of cancer. Thus, cancer cells with mutations in the causative genes specifically cause cell death, whereas normal cells rarely exhibit cell toxicity [11, 12]. Synthetic lethal genes for cancer-causing genes have been identified in many cancers [7–9, 13–15]. In particular, the clinical use of anticancer drugs developed using synthetic lethal phenotypes, such as PARP inhibitors for BRCA 1/2 mutated cancers, is advancing [16–18].

The Hippo pathway is a signaling pathway that regulates cell growth, organ size, and tissue regeneration [19, 20]. The central kinase in the Hippo pathway, LATS kinase, consists of two paralogs, LATS1 and LATS2 kinase. The functions of these two kinases are partly redundant and different [21]. LATS kinases phosphorylate transcriptional co-activators YAP1 and TAZ and inhibit their nuclear translocation [22]. The inability of YAP and TAZ to localize in the nucleus suppresses the expression of genes controlled by TEAD [23]. LATS2 is also involved in DNA damage response. When LATS2 is overexpressed, it phosphorylates c-Abl, thereby protecting cells from apoptosis caused by DNA damage [22]. However, when LATS2 is knocked down, it cannot phosphorylate c-Abl, and the cells undergo apoptosis [22]. In MM, LATS2 is a causative gene, and its decreased expression causes abnormal cell proliferation. However, no detailed molecular mechanisms underlying carcinogenesis have been reported [24, 25].

SMG6 (also known as EST1A) functions in NMD, DNA repair, and telomere maintenance [26]. In the NMD pathway, SMG6 cooperates with other NMD core factors, such as UPF1 (DNA-RNA helicase) and SMG1(PI3-related kinase), to degrade mRNA with an immature stop codon. UPF1 has been reported to be involved in DNA damage checkpoint activation and homologous recombination [27, 28]. SMG1 and SMG6 regulate the phosphorylation and dephosphorylation of UPF1, respectively, and thus may be involved in the DNA damage repair pathway, although direct reports have not yet been provided. In addition to NMD, SMG6 interacts with hTERT to remove telomere capping to maintain telomere integrity [29–31].

hTERT exhibits two enzymatic activities: RdDP and RdRP. hTERT functions as a RdDP that elongates telomeres from the RNA template TERC and maintains telomere homeostasis. SMG6 is important for recruitment to telomere ends and the tethering of telomerase [32]. Conversely, when hTERT functions as an RdRP, it binds to the non-coding RNA RMRP and synthesizes a double-stranded RNA that is a precursor of small interfering RNA [30]. The level of hTERT expression varies among cancer types. hTERT may be a potential anticarcinogenic target [33, 34].

In this study, we found that the downregulation of SMG6 induced a synthetic lethal phenotype in cell lines in which LATS2 was inactivated. This synthetic lethality requires the nuclear translocation of YAP and TAZ, downstream factors of LATS2 in the Hippo pathway. Since SMG6 regulates hTERT [35–40], inhibition of hTERT also resulted in a synthetic lethal phenotype in cell lines in which LATS2 was downregulated. We then demonstrated that hTERT RdDP activity was required to induce a synthetic lethal phenotype. Furthermore, this synthetic lethal phenotype has been confirmed in vivo. These results indicate that the Hippo pathway is associated with TERT activity via SMG6. We also propose SMG6 and TERT as novel molecular target candidates for LATS2-mutated MM.

## Results

### Cells with suppressed LATS2 expression are resistant to DNA damaging reagents

**Fig. 1:**
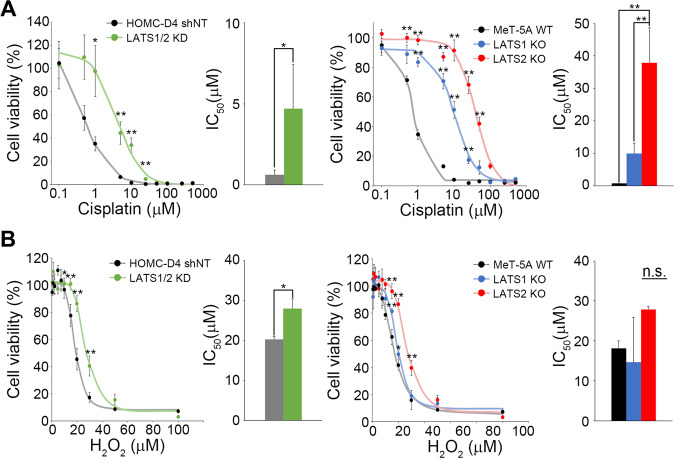
LATS2-inactive cells showed cisplatin resistance. Effect of cisplatin (**A**) and H_2_O_2_ (**B**) on LATS1/2 KD HOMC-D4 and LATS1 or LATS2 KO MeT-5A cells. The cells were treated with various concentrations of cisplatin or hydroperoxide for 24 h. After the medium change, the cells were cultured for an additional 48 h, and then cell viability was measured using CCK-8. All experiments were performed at least three independent times. Data are presented as means ± SD, and *p* values were calculated using the Tukey-Kramer method. * indicates *p* < 0.05 and ** indicates *p* < 0.01. n.s. means no significant difference.

Cisplatin and pemetrexed are currently used as standard treatments for MM, although the response rates are not very good [41, 42]. This suggests that mutations in genes responsible for MM may be involved in the DNA damage repair pathway. It has been reported that there is a correlation between the Hippo pathway and cisplatin resistance in ovarian and lung cancer and oral squamous cell carcinoma [43, 44]. We first prepared LATS1/2 knockdown (KD) immortalized mesothelial HOMC-D4 cells using lentivirus to determine whether LATS1/2 is also involved in DNA damage repair in mesothelial cells. Then, we examined whether they showed cisplatin resistance. As shown in Fig. 1A (left), the cytotoxicity of cisplatin was reduced in LATS1 and LATS2 double-KD mesothelial cells. To determine whether LATS1 or LATS2 is more involved in cisplatin resistance, we performed a similar experiment using immortalized mesothelial MeT-5A cells and MeT-5A cells in which LATS1 and LATS2 were knocked out, respectively. Although both LATS1 and LATS2 KO cells showed resistance to cisplatin, LATS2 KO cells were more resistant to cisplatin than LATS1 KO cells (Fig. 1A, right). Next, we examined whether LATS1 and/or LATS2 were involved in DNA single-strand break (SSB) repair using hydrogen peroxide. We found that LATS 1/2 KD cells were slightly more resistant to hydrogen peroxide than the control cells (Fig. 1B, left). We then treated LATS1 KO and LATS2 KO cells with hydrogen peroxide to examine their sensitivity. LATS1 KO cells showed almost the same sensitivity as control cells, whereas LATS2 KO cells were slightly more resistant than control cells (Fig. 1B, right). These results indicated that LATS2 kinase plays a more important role than LATS1 kinase in DNA double-strand break repair, as reported in other cell types [45]. In contrast, LATS kinases are less involved in SSB repair in mesothelial cells.

### LATS2-inactive cells exhibit a synthetic lethal phenotype by inhibition of SMG6

**Fig. 2:**
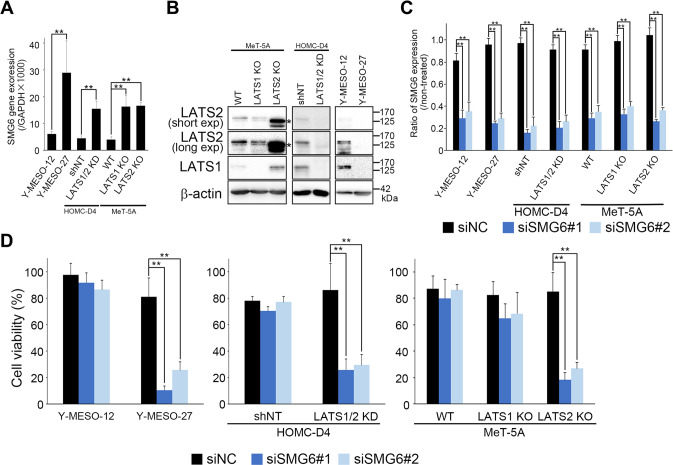
Knockdown of SMG6 reduces the viability of LATS2-inactive cells. **A** The mRNA expression levels of SMG6. **B** The protein expression levels of LATS1 and LATS2. * indicates a nonspecific band. **C** The knockdown efficiency of SMG6 mRNA. **D** Cell viability when SMG6 is knocked down. Cell viability was measured 72 h after siSMG6 treatment using CCK-8. All experiments were performed at least three independent times. Data are presented as means ± SD, and *p* values were calculated using the Tukey-Kramer method. ** indicates *p* < 0.01.

To elucidate why LATS2-inactive mesothelial cells acquire resistance to DNA damage, we focused on SMG6 because it is a factor related to DNA repair and integrity monitoring, and its expression is elevated in LATS2-inactive mesothelial cells (Fig. 2A). To this end, in addition to immortalized mesothelial cell lines, we also studied two mesothelioma cell lines (Y-MESO-12 and 27). Y-MESO-27 was a cell line in which both LATS1/2 were not expressed (Fig. 2B, Supplementary Fig. 9). YAP, pYAP, and TAZ expression in each cell line are shown in Fig. S1C. We then examined the effect of SMG6 knockdown on the viability of these cell lines. Although the knockdown efficiency of SMG6 did not differ significantly among cells (Figs. 2C, S1A), cell viability was dramatically reduced in Y-MESO-27 cells (Fig. 2D, left). The inactivation of LATS kinases decreased the viability of HOMC-D4 cells (Fig. 2D, middle). In MeT-5A cells, knocking out LATS2 decreased cell viability more than knocking out LATS1 (Fig. 2D, right). These results indicated that the knockdown of SMG6 reduces the viability of LATS2-inactive cells, indicating a synthetic lethal phenotype.

### Knockdown of SMG6 induced DNA damage and subsequent apoptosis in LATS2 KO cells

**Fig. 3:**
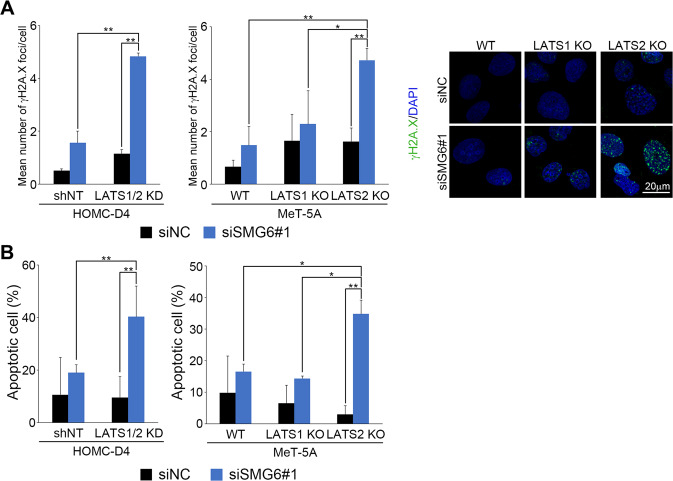
Knockdown of SMG6 induces DNA damage and apoptosis in LATS2-inactive mesothelial cells. **A** γ-H2A.X immunofluorescence and DAPI staining in various cells infected with control or SMG6-specific siRNA (siSMG6#1). The number of γ-H2A.X foci in each cell was counted 72 h after siSMG6 treatment. γ-H2AX is green, and DAPI is blue. **B** The number of apoptotic cells was measured by flow cytometry using APO-Direct Kit 72 h after siSMG6 treatment. All experiments were performed at least three independent times. Data are presented as means ± SD; *p*-values were calculated using the Tukey-Kramer method. * indicates *p* < 0.05 and ** indicates *p* < 0.01.

To investigate the cause of synthetic lethality, we analyzed SMG6 knockdown cells. First, the amount of DNA damage was measured using gamma-H2AX (γ-H2AX) staining. The number of γ-H2AX foci increased in LATS 1/2 KD HOMC-D4 cells treated with siSMG6 (Fig. 3A, left and Fig. S2A, left). Further investigation showed that when SMG6 was knocked down, the number of γ-H2AX foci increased in LATS2 KO cells but not in LATS1 KO MeT-5A cells (Fig. 3A, middle and right, Fig. S2A, right). The number of apoptotic cells was determined by TUNEL staining. TUNEL analysis revealed that SMG6 knockdown significantly increased the number of apoptotic LATS 1/2 KD cells (Fig. 3B, left and Fig. S2B, left). Notably, we found that LATS2-KO MeT-5A cells showed significantly more apoptotic cells than LATS1 KO cells (Fig. 3B, right; Fig. S2B, left). These results indicated that SMG6 knockdown induces apoptosis following DNA damage in LATS2 KO cells but only modestly in LATS1 KO cells.

### YAP1/TAZ activation is required for a synthetic lethality by SMG6 knockdown

To clarify the molecular mechanism of synthetic lethality induced by the co-suppression of LATS2 and SMG6, we analyzed the downstream factors involved in LATS2 and SMG6.

**Fig. 4:**
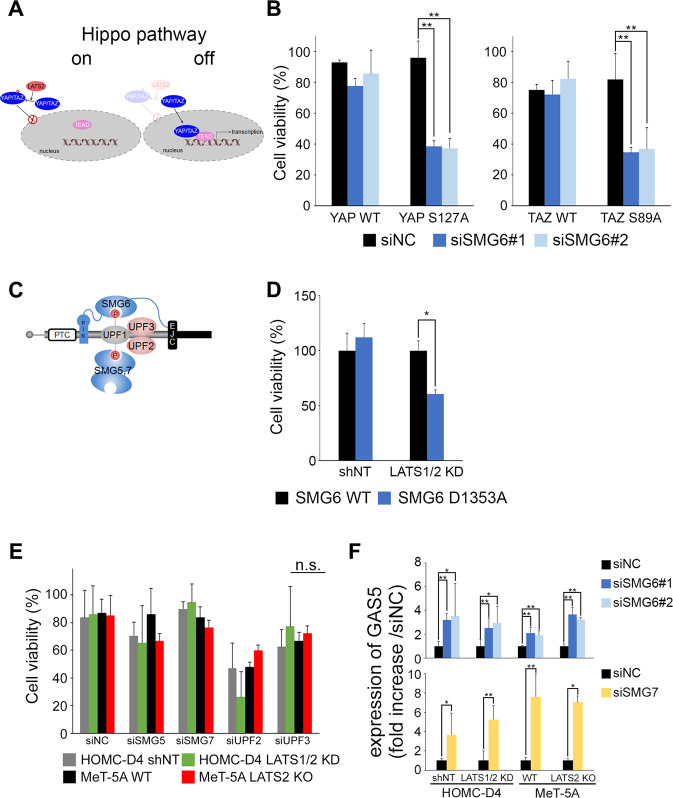
YAP/TAZ activity was required to induce synthetic lethality but not related to the NMD pathway factors. **A** Schematic drawing of Hippo pathway. **B** Non-phosphorylated forms of YAP/TAZ-overexpressing HOMC-D4 were transfected with siSMG6. Cell viability was measured after 144 h of siSMG6 treatment using CCK-8. **C** Schematic drawing of NMD complex. **D** (SMG6 WT and D1353A)-overexpressing plasmids were transfected into HOMC-D4 (non-target (shNT) and LATS1/2 KD cells. After 144 h of transfection, the cell viability was measured using CCK-8. **E** The viability of LATS2-inactive mesothelial cells treated with siSMG5, siSMG7, siUPF2, or siUPF3. Cell viability was measured using CCK-8. **F** The expression levels of GAS5 in siSMG6- (upper panel) or siSMG7- (lower panel) treated LATS2-inactive mesothelial cells. All experiments were performed at least three independent times. Data are presented as means ± SD, and *p* values were calculated using the Tukey-Kramer method. * indicates *p* < 0.05 and ** indicates *p* < 0.01. n.s. - no significant difference. ND means not detect.

LATS2 phosphorylates YAP1 and its paralog TAZ in the Hippo pathway, thereby inhibiting their entry into the nucleus and repressing transcription by TEADs (Fig. 4A). We, therefore, examined whether the active YAP/TAZ is involved in the synthetic lethal phenotype by inhibiting SMG6 in cells overexpressing the non-phosphorylated variants YAP S127A and TAZ S89A (Fig. S6B, S9). Furthermore, knockdown of SMG6 decreased the viability of cells overexpressing YAP S127A and TAZ S89A (Fig. 4B).

This result indicated that the synthetic lethal phenotype induced by the co-reduction of LATS2 and SMG6 requires YAP1/TAZ activation.

### Factors in the NMD pathway other than SMG6 are not involved in induction of synthetic lethality in LATS2 KO cells

One of the key functions of SMG6 is the regulation of NMD during the degradation of immature mRNAs. SMG5 and SMG6, members of the functional complex of NMD, contain PIN domains, but only SMG6 has ribonuclease activity (Fig. 4C). The D1353 residue in the PIN domain of SMG6 is an important active site [46]. We then examined cell viability when SMG6 D1353A, the dominant-negative form of SMG6, was overexpressed in the LATS1/2 KD cells. As shown in Fig. 4D, overexpression of the dominant-negative form of SMG6 decreased the viability of LATS 1/2 KD cells. This suggested that the nuclease activity of SMG6 is required to induce synthetic lethality in LATS 1/2 KD cells.

Next, we examined whether NMD factors other than SMG6 (Fig. 4C) were involved in inducing synthetic lethality in LATS2-inactive mesothelial cells. Unexpectedly, the knockdown of the NMD factor did not reduce the viability of LATS2-inactive cells (Fig. 4E). However, the expression of a non-coding RNA growth arrest-specific 5 (GAS5) that was reported to be elevated following NMD suppression [47, 48] was elevated by SMG6 and SMG7 knockdown and NMD was inhibited (Fig. 4F).

These results indicated that the synthetic lethal phenotype induced by the co-reduction of LATS2 and SMG6 requires YAP1/TAZ activation but not the NMD pathway regulated by SMG6. However, the nuclease activity of SMG6 appears to be necessary for this synthetic lethality.

### Inactivation of LATS2 and inhibition of hTERT showed the synthetic lethal phenotype, and RdDP activity of hTERT is required for this phenotype

Another major role of SMG6 is in DNA repair associated with hTERT ligation to maintain telomere homeostasis [26, 49, 50]. Therefore, to investigate whether TERT is involved in the induction of synthetic lethality by the co-inhibition of LATS2 and SMG6, we examined cell viability when TERT was suppressed in LATS2-inactive MM cells.

**Fig. 5:**
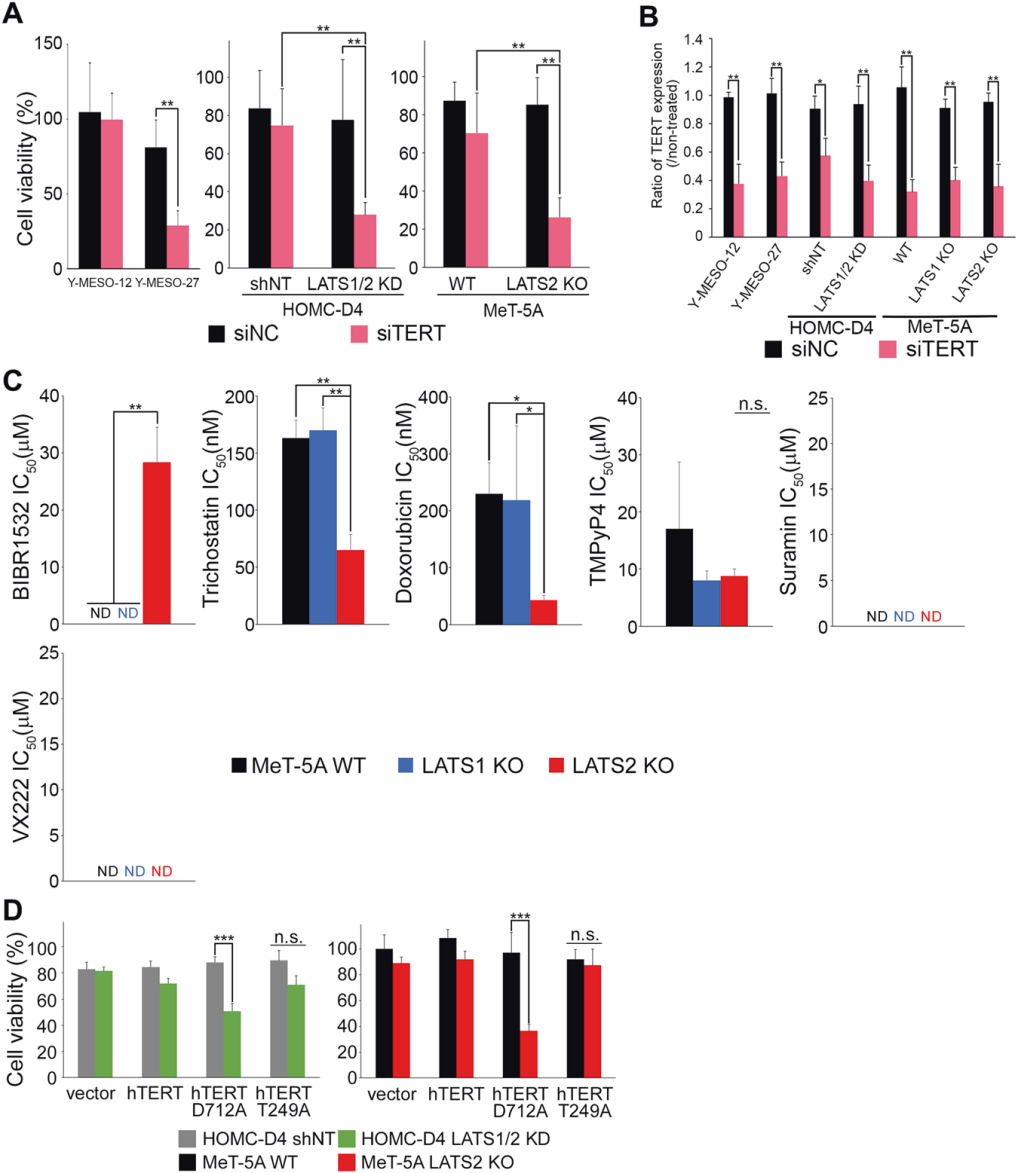
Inactivation of LATS2 and inhibition of TERT show a synthetic lethal phenotype, requiring RdDP activity of TERT. **A** Each cell was transfected with siTERT. Cell viability was measured after 144 h of siTERT treatment using CCK-8. **B** The knockdown efficiency of TERT. mRNA was collected after 72 h of siTERT treatment. **C** The IC_50_ of the TERT inhibitors (BIBR1532, trichostatin, doxorubicin, TMPyP4, suramin, and VX222) in MeT-5A (WT, LATS1 KO, and LATS2 KO). The diluted TERT inhibitor was applied to each cell, and cell viability was measured after 144 h of incubation using CCK-8. **D** hTERT D712A- and hTERT T249A-overexpressing plasmids were transfected into HOMC-D4 (non-target [shNT] and LATS1/2 KD) and MeT-5A (WT and LATS2 KO) cells. The cell viability was measured after 144 h of transfection using CCK-8. All experiments were performed at least three independent times. Data are presented as means ± SD, and *p* values were calculated using the Tukey-Kramer method. * indicates *p* < 0.05, ** indicates *p* < 0.01 and *** indicates *p* < 0.001. n.s. - no significant difference.

TERT knockdown significantly decreased the viability of Y-MESO-27 cells, in which LATS2 was inactivated (Fig. 5A left, B, and Fig. S1B). The viability of Y-MESO-12 cells expressing LATS2 was almost the same as that of control cells (Fig. 5A left, B, and Fig. S1B). Furthermore, TERT inhibition (Fig. 5B, Fig. S1B) reduced the viability of LATS2-inactive mesothelial cells (Fig. 5A, middle and right).

TERT has RNA-dependent DNA polymerase (RdDP) and RNA-dependent RNA polymerase (RdRP) activities [51]. To determine the activity involved in this synthetic lethality, we examined cell viability using TERT inhibitors in LATS1 KO and LATS2 KO cells. Fig. 5C shows the IC_50_ values of each drug in the tested cells. Among the hTERT inhibitors used, drugs with RdDP inhibitory functions, such as doxorubicin [52], trichostatin [53, 54], and BIBR 1532 [55], reduced the viability of only LATS2 KO cells in a dose-dependent manner (Figs. 5C, S3). In contrast, treatment with the RdRP-specific inhibitors TMPyP4 and VX 222 did not reduce the viability of any cell in the dose range tested (Figs. 5C, S3). Cell viability was examined by overexpressing hTERT D712A [56], a dominant-negative for RdDP, or T249A [34], a dominant-negative for RdRP, in LATS2-inactive mesothelial cells. Cell viability decreased only when hTERT D712A was overexpressed (Fig. 5D). These results suggested that the synthetic lethality caused by the suppression of SMG6 in LATS2 knockout cells is mediated mainly by TERT RdDP activity.

### Suppression of SMG6 expression and TERT inhibition reduce tumor growth in MM bearing mouse models

**Fig. 6:**
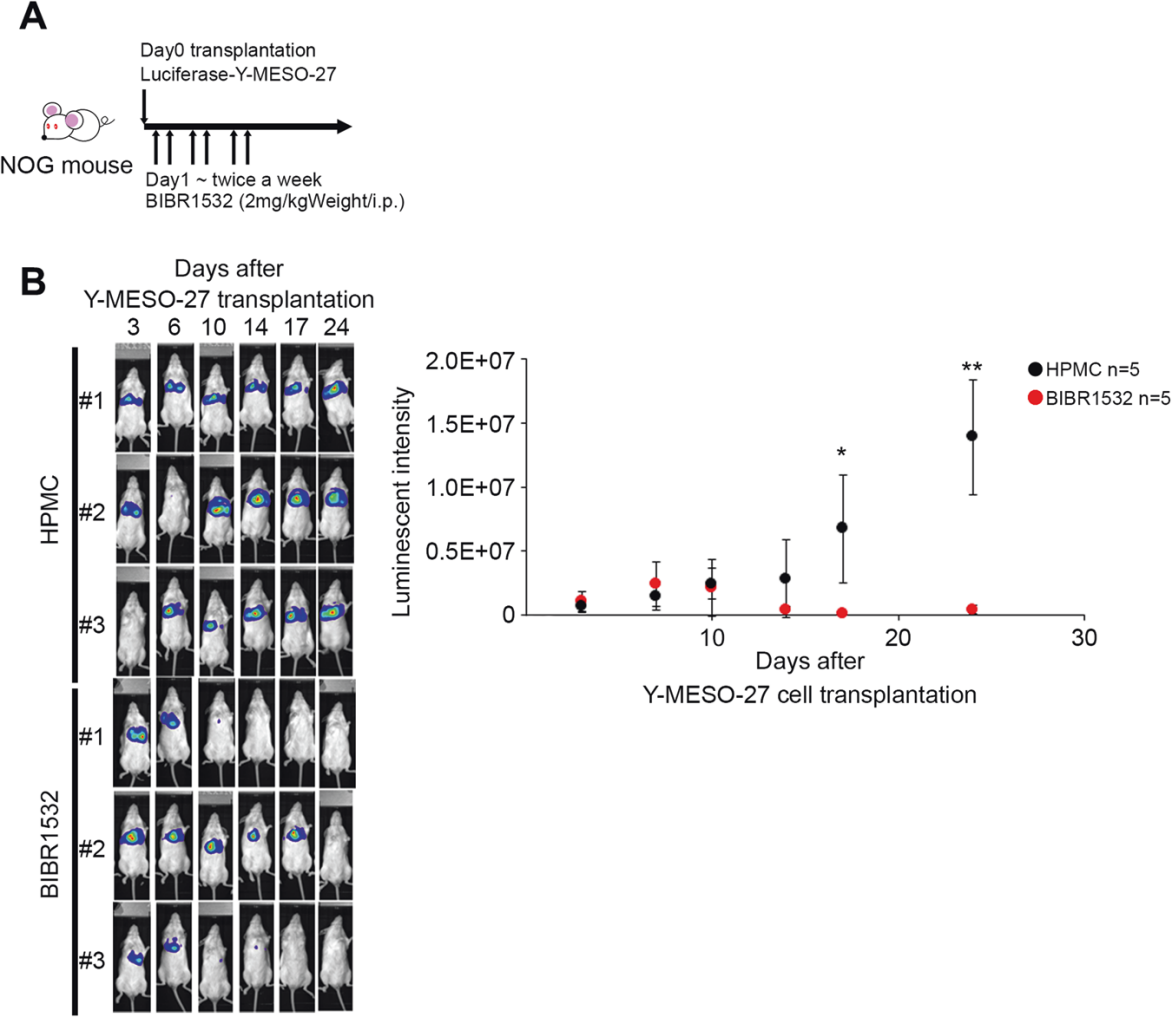
Synthetic lethality induced by inhibition of LATS2 and SMG6/TERT is also observed in a tumor-bearing mouse model. **A** Timetable for transplantation, drug administration, and IVIS measurements. **B** The IVIS images were obtained after tumor engraft subcutaneous inoculation of luciferase induced-Y-MESO-27 cells. One day after Y-MESO-27 transplantation, HPMC (*n* = 5) or BIBR1532 (2 mg/kg, *n* = 5) were injected in peritoneal cavity twice a week. Tumor size was measured by IVIS twice weekly after tumor transplantation until day 30. The luminescent intensity indicated the levels of quantification of IVIS imaging. *p* values were calculated using student’s *t*-test.* indicates *p* < 0.05 and ** indicates *p* < 0.01.

Finally, to assess LATS2-SMG6 synthetic lethality in vivo, we generated an MM-bearing mouse model and measured the tumor volume using the IVIS live imaging system. LATS2-mutated Y-MESO-27 cells were implanted into the mouse thoracic cavity and BIBR 1532 (2 mg/kg) was intraperitoneally administered twice-weekly after transplanting cells to investigate tumor growth and mouse survival (Fig. 6A). The tumor mass was significantly reduced by BIBR1532 after day17 in the Y-MESO-27 implanted mouse (Fig. 6B). Furthermore, when LATS2 KO cells were implanted with siSMG6, the luminescence intensity did not increase (Fig. S6A). In addition, the luminescence intensity decreased significantly after day 8 of BIBR 1532 treatment (Fig. S6B); that is, tumor growth was inhibited.

These results confirmed that the synthetic lethality induced by LATS2-SMG6/TERT observed in vitro also occurs in vivo, suggesting the possibility that TERT and SMG6 are effective against LATS2-mutated cancers such as MM.

## Discussion

This study found that silencing SMG6 expression in LATS2-inactive cells induced apoptosis following DNA damage, resulting in a synthetic lethal phenotype. The induction of synthetic lethality requires the Hippo pathway factors. In addition, the TERT regulatory function of SMG6 is required, but its role in NMD is not required. TERT inhibition also resulted in a synthetic lethal phenotype in LATS2-inactive cells. Furthermore, we found that TERT RdDP activity was required to induce this synthetic lethality. This phenotype was confirmed in vivo.

Possible clues to the molecular mechanism include the following: LATS2 inactivation increases p73 transcription by TEAD, and knockdown of SMG6 further increases p73 expression through ATM stabilization (Fig. S5A) [57, 58]. These synergistic effects induced apoptosis. Indeed, LATS2 KO cells tended to express more p73 than control cells (Fig. S5B, left), and knockdown of SMG6 tended to further increase p73 expression (Fig. S5B, left). In addition, p73 accumulated in the nuclei of SMG6-knockdown LATS2 KO cells (Fig. S5C).

In addition, ATM expression increased in all WT, LATS1 KO, and LATS2 KO cells upon the knockdown of SMG6 (Fig. S5B left). SMG6 is also involved in DNA damage repair, and further DNA damage caused by SMG6 knockdown may result in apoptosis (Fig. 3B). The involvement of SMG6 in DNA repair has not yet been directly demonstrated [59–61]. However, it has been reported that SMG1 phosphorylates p53 during DNA damage and that knockdown of SMG1 increases DNA damage and radiation sensitivity. Given that SMG1 and SMG6 both regulate UPF1 phosphorylation, it is likely that SMG6 is involved in DNA repair [62]. The SMG6/TERT complex promotes telomere elongation, thereby maintaining telomere ends protected by TRF1,2 and preventing apoptosis (Fig. S5D) [63, 64]. Knockdown of SMG6 in LATS 1KO and 2 KO cells also decreased TRF1 and TRF2 expression (Fig. S5E) and increased DNA damage (Fig. 3A). However, increased DNA damage did not co-localize with TRF2 (Fig. S5F); therefore, SMG6 may be involved in DNA damage repair in addition to telomere-end maintenance.

We found that SMG6 expression is upregulated in LATS-inactivated cells (Fig. 2A) and that loss of SMG6 induces cell death in LATS2-inactivated cells and in cells overexpressing the active form of YAP/TAZ (Figs. 2D, 4B). This suggests that SMG6 is a target of YAP/TAZ. Therefore, we searched the ChEA tool [65], which was constructed by integrating ChIP-chip, ChIP-seq, ChIP-PET, and DamID, using Harmonizome (https://maayanlab.cloud/Harmonizome/) [66]. This search identified SMG6 as a target of YAP1 (data not shown). In addition, we observed higher SMG6 expression in HOMC-D4 cells overexpressing YAP S127A or TAZ S89A than in cells overexpressing wild-type YAP or TAZ (Fig. S6A).

YAP has been reported to positively regulate TERT expression in the human liver and lung cancer cells [67, 68]. Therefore, LATS2 mutations in MM may result in increased expression of non-phosphorylated forms of YAP and TAZ, which can enter the nucleus, leading to increased expression of their regulated genes, including TERT, and increased cellular dependence on TERT. Therefore, TERT knockdown might induce cell death.

However, the molecular mechanism by which the inhibition of RdDP activity of TERT causes DNA damage in the whole nucleus in LATS2 KO cells remains unknown. The involvement of the nuclease activity of SMG6 in this phenotype is also unknown. Further studies are needed on these.

NF2, which is upstream of LATS2 kinase in the Hippo pathway, is another causative gene for malignant mesothelioma [69]. We investigated whether inhibiting SMG6 in cells with NF2 mutations would also induce a synthetic lethal phenotype. SMG6 knockdown reduced cell viability in H2052 and H2373 cells (Fig. S7A). Further investigation is needed to clarify the relationship between NF2 mutations and SMG6/TERT expression.

As no inhibitors of SMG6 have been marketed to date, NMDI (an inhibitor of SMG7), known as an NMD inhibitor, was investigated. Treatment of the LATS2 mutant cell line with NMDI did not alter cell viability (unpublished data). This is consistent with the results shown in Fig. 4E. Therefore, when SMG6 is used as a molecular target for MM, it may be necessary to consider, for example, nucleic acid drugs. From the results of the crystal structure analysis, we extracted candidate pocket-like sites that could bind small molecules in SMG6 and may inhibit the activity of SMG6 (Fig. S8A). The pocket site was located within the PIN domain. We constructed the expression vector of SMG6 with a mutation of the amino acid residue. This is important for pocket formation (R1407, F1412, and W1415). The viability of LATS 1/2 KD cells overexpressing the pocket site variant of SMG6 was examined. Overexpression of R1407 A and W1415 A significantly decreased the viability of LATS 1/2 KD cells, but there was no significant difference in viability between the control cells and cells overexpressing F1412 A (Fig. S8B). This may be because F1412 is located near the RNA-binding pocket and is not involved in the formation of the binding pocket. Since it has been reported that the W1415 site is involved in RNA binding [46], this may provide a clue that the nuclease activity of SMG6 is required for the synthetic lethal phenotype. Alternatively, the binding site of SMG6 to TERT has not yet been reported [37], but this pocket may be involved in binding. Administration of a TERT inhibitor inhibited LATS2-mutated tumor growth in tumor-bearing mouse models. It may be a novel target for LATS2-mutated MM and LATS2-mutated cancer.

In addition to elucidating the molecular mechanisms, further detailed studies using in vivo models are needed to obtain novel therapeutic candidates for LATS2-mutated MM.

## Materials and methods

### Ethics statement

All animal experiments were performed following protocols approved by the Animal Studies Committee of Juntendo University (the Ethics Committee for Animals at Juntendo University No. 1401). Recombinant DNA experiments were approved by the Committee for Recombinant DNA Experiments at Juntendo University (No. DNA30-69), Tokyo University of Technology (No. 20-BS-01-067, No. 20-BS-02-068), and the Aichi Cancer Center Research Institute (No. 2208). Establishment of cell lines was approved by the Ethics Committee at the Aichi Cancer Center (No. 19-12), and written informed consent was obtained from the patients.

### Cell lines and cell culture

The human immortalized mesothelial cell line MeT-5A (CRL-9444) and human malignant mesothelioma cell lines (NCI-H2052 and NCI-H2373) were purchased from ATCC. HOMC-D4 [70], YMESO-12, -27 were established by Dr. Sekido. HOMC-D4, Y-MESO-12, and Y-MESO-27 cell lines have been deposited to RIKEN BioResource Research Center (Tsukuba, Japan). All cell lines were cultured in RPMI-1640 medium supplemented with 10% fetal bovine serum (FBS) and 1% antimicrobial at 37 °C in a humidified incubator with 5% CO2. All cell lines were Mycoplasma-free and were identified using short-tandem repeat analysis or a single nucleotide polymorphism array.

HOMC-D4 that overexpress TAZ WT or TAZ S89A ^42^were established by Dr. Sekido. MeT-5A cell lines with the knockouts of LATS1 or LATS2 were also established (described elsewhere). HOMC-D4 non-target shRNA control and LATS1 and LATS2 knockdown cells were generated in this study. HOMC-D4 cells that overexpress YAP WT or YAP S127A were generated in this study. The shRNA plasmids used are listed in Table 1. The expression plasmids used are listed in Table 2.

**Table 1:** List of primers for real-time qPCR, cloning, and sequencing.

real-time qPCR		
Probe	Forward primer	Reverse primer
hGAPDH	CTCCTCCACCTTTGACGCT	TTGCTGTAGCCAAATTCGTT
hSMG6	ATGGGAAAGGAAATGGGAAG	CTGCCTTCAGCCTCTGAATC
hGAS5	GCACCTTATGGACAGTTG	GGAGCAGAACCATTAAGC
hTERT	CGTACAGGTTTCACGCATGTG	ATGACGCGCAGGAAAAATG
hTRF2	CCCAAGAACAAGCGCATGAC	TTTCTGCACTCCAGCCTTGAC

cloning		
Probe	Forward primer	Reverse primer
SMG6 WT BamH1	GGATCCatggcggaagggctggagcg	GTCGACTCAtcagcccacctgggcccac
SMG6 D1353A	gctgatctcatcctgtcctg	gttgttacccagctggccag
SMG6 R1407A	cctgtaGCTgacatcccagccttcctc	gatgtcAGCtacaggaacattccttgt
SMG6 F1412A	ccagccGCCctcacgtgggcccaggtg	cgtgagGGCggctgggatgtcccgtac
SMG6 W1415A	ctcacgGCTgcccaggtgggctga	ctgggcAGCcgtgaggaaggctgggat

sequence		
probe		sequence
SMG6	sequence1	GGTGGAGGAGGAAGAAGTC
	sequence2	CCAGGAGAAGTTCAGAAAGG
	sequence3	GATGATGAAGTCAGCCCTAC
	sequence4	GGTGATTGAGAAGTTCAGG
	sequence5	GCGGAAAGGAAAGAAGTCTAC
	sequence6	CAAGGTGTCTTCCTTTGTC
	sequence7	GAGGATGACATCAGGGAGC
	sequence8	CCTCCACTACTGCAAAGAC

siRNA			
probe	sense	antisense	siRNA ID#
SMG6 #1	GGGUCACAGUGCUGAAGUAtt	UACUUCAGCACUGUGACCCtt	24921
SMG6 #2	GUCUAUUACUAUAUGCGCAtt	UGCGCAUAUAGUAAUAGACAG	s23488
TERT	GCACGGCUUUUGUUCAGAUtt	AUCUGAACAAAAGCCGUGCCA	s370
SMG5	CGACUUGACCUCAUCCUUUtt	AAAGGAUGAGGUCAAGUCGat	s23695
SMG7	GGCUGAUAACAGAUCUGUAtt	UACAGAUCUGUUAUCAGCCat	s19152
UPF2	GAGGUUCCAGUAAACGAAAtt	UUUCGUUUACUGGAACCUCtg	s24946
UPF3	GGUGGUAAUUCGAAGAUUAtt	UAAUCUUCGAAUUACCACCtt	s35213
Negative Control	Invitrogen Silencer Select siRNA Negative Control #1 (undisclosed)		439084

shRNA	
probe	sequence
shLATS1 #1267	CCGGAACATTAGTGTACCTGGACTCGAGCAGTCCAGGTACACTAATGTTTTTTTTG
shLATS2 #0207	CCGGAAGTTCGGACCTTATCAGAAACTCGAGTTTCTGATAAGGTCCGAACTTTTTTTTG

**Table 2:** List of plasmids.

Lentiviral Vector	psPAX2	Addgene
	pMD2.G	Addgene
	pLKO.1 shNontarget	in this study
	pLKO.1 shLATS1	in this study
	pLKO.1 shLATS2	in this study
	CRISPR LATS1 gRNA	in this study
	CRISPR LATS2 gRNA	in this study
	pLKO-CMV-EGFP-TAZ WT	A Matsushita et al.
	pLKO-CMV-EGFP-TAZ S89A	A Matsushita et al.
	pLKO-CMV-EGFP-YAP1 WT	in this study
	pLKO-CMV-EGFP-YAP1 S127A	in this study
Expression vector	pCMV-SMG6 WT	in this study
	pCMV-SMG6 D1353A	in this study
	pCMV-SMG6 R1407A	in this study
	pCMV-SMG6 F1412A	in this study
	pCMV-SMG6 W1415A	in this study
	pBABE-TERT WT	M Yasukawa et al.
	pBABE-TERT D712A	M Yasukawa et al.
	pBABE-TERT T249A	M Yasukawa et al.
Retroviral Expression system	MSCV-YAP WT-IRES-GFP	T Kakiuchi et al.
	MSCV-YAP S127A-IRES-GFP	T Kakiuchi et al.

### siRNAs, plasmids, and transfections

Silencer Select siRNAs for SMG6 (Cat No. 24921, s23488), SMG5 (Cat No. S23695), SMG7 (Cat No. S19152), UPF2 (Cat No. S24946), UPF3 (Cat No. S35213), TERT (Cat No. S370, s371), and negative control (Cat No. 4390843) were purchased from Thermo Fisher Scientific. The expression vectors pBp-hTERT WT, D712A, and T249A were provided by Dr. Masutomi [34, 71]. pCMV-SMG6 WT, R1407A, F1412A, and W1415A plasmids were constructed in this study. Lentiviral vectors (pLKO-CMV-EGFP-TAZ1 WT/S89A and pLKO-CMV-EGFP-YAP1 WT/S127A) were constructed by Dr. Sekido [72]. SMG6 was cloned into pCMV-Myc from cDNA derived from HeLa cells. A plasmid expressing SMG6 without nuclease activity (SMG6 D1353A) was prepared by performing inverse PCR using the primers shown in Table 1 [73].

siRNA was transfected into each cell using Lipofectamine RNAiMax Transfection Reagent (Thermo Fisher Scientific, Massachusetts, USA;Cat No. 13378150). Cloned plasmids were transfected into each cell using Lipofectamine 3000 (Thermo Fisher Scientific, Cat No. L3000008). After transfection, the medium was changed after 24 h, and the cells were cultured in a culture medium.

### Reagents

Hydrogen peroxide (Cat No.081-04215), DMSO (Cat No. 3176), and cisplatin (Cat No. 039-20093) were purchased from FUJIFILM Wako Pure Chemical (Tokyo, Japan). Inhibitor reagents targeting hTERT (BIBR1532; Cat No. SC-20843) [55], trichostatin (Cat No. SC-3511) [74], doxorubicin (Cat No. SC-200923) [75], TMPyP4 (Cat No. SC-204346), and suramin (Cat No. SC-200833) were purchased from Santa Cruz Biotechnology (California, USA). The RdRP-specific inhibitor VX-222 (Cat No. S1480) was purchased from Selleck Chemicals (Houston, USA). HPMC (Cat No. 44779) was purchased from Alfa Aesar (Massachusetts, USA).

### Cell viability assay

Cell viability and viability curves were analyzed using the Cell Counting Kit-8 (CCK-8) (FUJIFILM Wako Pure Chemical Corporation, Cat No. 341-08001) following the manufacturer’s instructions.

### Reverse transcription and quantitative PCR

Total RNA was isolated using ISOSPIN cells and tissue RNA (FUJIFILM Wako Pure Chemical Corporation, Cat. No. 314-08211), and cDNA was synthesized using ReverTraAce (TOYOBO, Osaka, Japan; Cat No. TRT-101) following the manufacturer’s instructions. Quantitative real-time polymerase chain reaction (qPCR) was performed using a KAPA SYBR Fast qPCR Kit (NIPPON Genetics, Tokyo Japan; Cat. No. KK4602) and the 7900HT Fast Real-Time PCR System (Applied Biosystems, Tokyo, Japan). Primers used for qPCR are listed in Table 1.

### Western blotting

Cells were lysed in lysis buffer (150 mM NaCl, 0.1% Triton-X100, 0.5% Deoxycholic acid, 0.1% sodium dodecyl sulfate [SDS], 50 mM Tris-HCl pH8.0) and centrifuged at 12,000 g for 20 min at 4 °C. The supernatant was mixed with sample buffer (3% SDS, 6% glycerol, 40 mM tris-HCl pH 7.0, 0.01% bromophenol blue, 1.5% 2-mercaptoethanol) and boiled for 10 min at 95 °C. SDS-PAGE and western blotting were used to analyze cell lysates. Blots were detected using ECL Plus (GE Healthcare, Chicago, USA; Cat No. RPN2124) following the manufacturer’s protocol. The antibodies used are listed in Table 3.

**Table 3:** List of antibodies.

Epitope	Company	Catalog Number	conjugated
γH2A.X	Sigma	05-636	no
TRF2	BETHYL	A300-796A	no
LATS1	Cell signaling technology	91535	no
LATS2	Abnova	PAB4559	no
YAP	Cell signaling technology	14074	no
pYAP	Cell signaling technology	4911	no
TAZ	Thermo Fisher Scientific	PA5-72579	no
p73(5B429)	Novus Biologicals	NBP2-24737	no
pATM	Abcam	ab81292	no
β-actin	Cell signaling technology	4970 T	no
mouse IgG	Invitrogen	A28175	Alexa488
rabbit IgG	Invitrogen	A11035	Alexa546

### Crystal structure and 3D models of protein

The human SMG6 PIN domain structure was generated using PyMOL (PDB accession number: 2HWW).

### Immunofluorescence staining

The cells were seeded onto glass coverslips. The cells were fixed in cold methanol for 10 min at −20 °C and blocked with 1% BSA-PBS for 30 min at room temperature. Subsequently, the samples were immersed in the primary antibody overnight at 4 °C. The samples were stained with fluorescently labeled secondary antibodies with 1 mg/mL 4’,6-diamidino-2-phenylindole (DAPI) for 1 h at room temperature and embedded in a fluorescence mounting medium (Dako Japan, Tokyo, Japan; Cat. No. S3023). The antibodies used are listed in Table 3.

### Microscopy

Immunofluorescence images were captured and/or tiled using the confocal laser scanning microscope LSM780 (Carl Zeiss Japan). The acquired images were analyzed using ZEN 2012 (Carl Zeiss, Jena, Germany). Bright-field images were captured and/or tiled using an all-in-one microscope BZ-X700 (KEYENCE Japan). The acquired images were analyzed using a BZ-X analyzer (KEYENCE, Japan).

### Flow cytometry

Apoptotic cells were detected using the APO-DIRECT Kit (BD Biosciences Japan, Cat No. 556381) following the manufacturer’s instructions. The cell suspension was examined using a BD FACS Canto II system (BD Biosciences, Japan). The acquired data were analyzed using FlowJo software (FlowJo LLC).

### In vivo experiments

Six-week-old female NOD/Shi-scid IL-2Rγnull (NOG) mice purchased from Vivo Science were maintained under specific pathogen-free conditions. After anesthetization, 1 × 10^6^ luciferase-expressing YESO-27, MeT-5A LATS2 KO siNC, or siSMG6 cells were injected into the right thoracic cavity [76]. Each mouse was intraperitoneally injected with 5 μmol/mouse luciferin to assess tumor development. Ten minutes after luciferin i. p. administration, luminescence was quantified using the IVIS^TM^ imaging system (Summit Pharmaceuticals, Tokyo, Japan).

### Statistical analysis

All data are expressed as the mean ± SD. Comparisons between two groups were made using the Student’s t-test. Differences were considered statistically significant at *p* < 0.05. Comparisons among more than three groups were analysed using one way ANOVA on ranks, followed by the Tukey-kramer method carrying out Statcel4 software (OMS Publishing, Tokyo, Japan).

## Supplementary information

Supplemental Figure Legend

Supplemental Figure 1

Supplemental Figure 2

Supplemental Figure 3

Supplemental Figure 4

Supplemental Figure 5

Supplemental Figure 6

Supplemental Figure 7

Supplemental Figure 8

Supplemental Figure 9

Confirmation the author names

## Data Availability

All data are available in the main text or the supplementary materials.
