# Supplementary material for: SMG6 regulates DNA damage and cell survival in Hippo pathway kinase LATS2-inactivated malignant mesothelioma
Source: Cell Death Discov. 2022 Nov 5;8:446. doi: 10.1038/s41420-022-01232-w (PMC9637146; doi:10.1038/s41420-022-01232-w)
Supplement: Supplementary file 1 — Supplemental Figure Legend [file 41420_2022_1232_MOESM1_ESM.docx]

**Figure S1**.

(A-B) The mRNA expression levels of SMG6 (A) and TERT (B) 72 hours after transfection with SMG6 siRNA at various concentrations (0.5, 1, 2.5, 5, and 10 nM). The experiments in the text were performed with 5 nM siRNA. (C) YAP, pYAP, and TAZ expression in cells used in this study.

**Figure S2.**

γ-H2A.X immunofluorescence and DAPI staining in various cells infected with control or SMG6-specific siRNA (siSMG6#2). The number of γ-H2A.X foci in each cell nucleus was counted 72 h after treatment with siSMG6.

(B) The number of apoptotic cells was measured by flow cytometry using APO-Direct Kits 72 h after treatment with siSMG6.

**Figure S3.**

The viability of MeT-5A (WT, LATS1 KO, and LATS2 KO) cells treated with TERT inhibitors (BIBR1532, trichostatin, doxorubicin, TMPyP4, suramin, or VX222). The serially diluted TERT inhibitors were applied to cells, and the media were changed twice during the 144-hours incubation, followed by measuring cell viability using CCK-8. Data are presented as means ± SD. The p values were caluculated by the Tukey-kramer method. * indicates *p* < 0.05 and ** indicates *p* < 0.01.

**Figure S4.**

The IVIS images were obtained after tumor engraft subcutaneous inoculation of luciferase induced-MeT-5A LATS2 KO cells. (A) siNC (n = 5) or siSMG6 (n = 5) were transfected in LATS2 KO cells just before transplantation. (B) DMSO (n = 5) or the BIBR1532 (2 mg/kg, n = 5) were treated once every two days after transplantation. IVIS was used to measure tumor size every 2 days after tumor implantation until day 10. The luminescent intensity indicated the levels of quantification of IVIS imaging. Data are presented as means ± SD. *P* values were calculated by the the Tukey-kramer method. * indicates *p* < 0.05 and ** indicates *p* < 0.01.

**Figure S5.**

(A) Schematic diagram of transcription of p73 by the Hippo pathway and stabilization of p73 by ATM.

(B) Relative p73 and ATM expression in MeT-5A (WT, LATS1 KO, and LATS2 KO) cells treated with negative control (siNC) or siSMG6#1. mRNA was isolated 72 hours after siRNA transfection. Each experiment was performed at least three times independently. Data are presented as means ± SD. *P* values were calculated by the the Tukey-kramer method. * indicates *p* < 0.05 and ** indicates *p* < 0.01.

(C) Immunofluorescence staining for p73, TRF2, and DAPI in (WT, LATS1 KO, and LATS2 KO) cells transfected with negative control (siNC) or siSMG6#1. TRF is green, p73 is red, and DAPI is blue. Cells were fixed using ice-cold methanol 72 h after transfection.

(D) Schematic diagram of telomere end and SMG6/TERT complex.

(E) Relative expressions of TRF1 and TRF2 in MeT-5A (WT, LATS1 KO, and LATS2 KO) cells treated with siNC or siSMG6#1. mRNA was corrected after 72 hours with siRNA transfection. Each experiment was performed at least three times independently. Data are presented as means ± SD. *P* values were calculated by the theTukey-kramer method. ** indicates *p* < 0.01.

(F) Immunofluorescence staining for TRF2, pATM, and DAPI in MeT-5A (WT, LATS1 KO, and LATS2 KO) cells transfected with negative control (siNC) or siSMG6#1. TRF2 is green, pATM is red, and DAPI is blue. Cells were fixed with ice-cold methanol 72 h after transfection.

**Figure S6.**

(A) SMG6 mRNA levels in wild type or unphosphorylated YAP/TAZ-overexpressing HOMC-D4 cells and parent HOMC-D4 cells. Data are presented as means ± SD. *p* values were calculated using the Tukey-kramer method. ** indicates *p* < 0.01.

(B) Wild type or unphosphorylated YAP/TAZ protein in YAP/TAZ-overexpressing HOMC-D4 cells.

**Figure S7.**

(A) Cell viability when SMG6 is knocked down in NF2 mutant cells (H2052 and H2373). Cell viability was measured 72 h after siRNA treatment using CCK-8 assays. Data are presented as means ± SD. *p* values were calculated using the Tukey-kramer method. ** indicates *p* < 0.01. n.s. - no significant difference.

**Figure S8.**

(A) Surface representation of the crystal structure depicting the pocket (magenta) of SMG6, including PIN domain. Protein Data Bank accession No. 2hww. W1415 and R1407 constructed at the pocket of SMG6 and F1412 were oriented near the pocket.

(B) (SMG6 WT, SMG6 R1407A, F1412A, and W1415A)-overexpressing plasmids were transfected into HOMC-D4 (non-target (shNT) and LATS1/2 KD) cells. The media was changed twice during the 144-hour culture; cell viability was measured using CCK-8 (right panel). Each experiment was performed at least three times independently. Data are presented as means ± SD. *P* values were calculated by the Tukey-kramer method. ** indicates *p* < 0.01.

**Figure S9.**

Full and uncropped western blots in this study.
