# Supplementary figures and images for: SMG6 regulates DNA damage and cell survival in Hippo pathway kinase LATS2-inactivated malignant mesothelioma
Source: Cell Death Discov. 2022 Nov 5;8:446. doi: 10.1038/s41420-022-01232-w (PMC9637146; doi:10.1038/s41420-022-01232-w)

**A**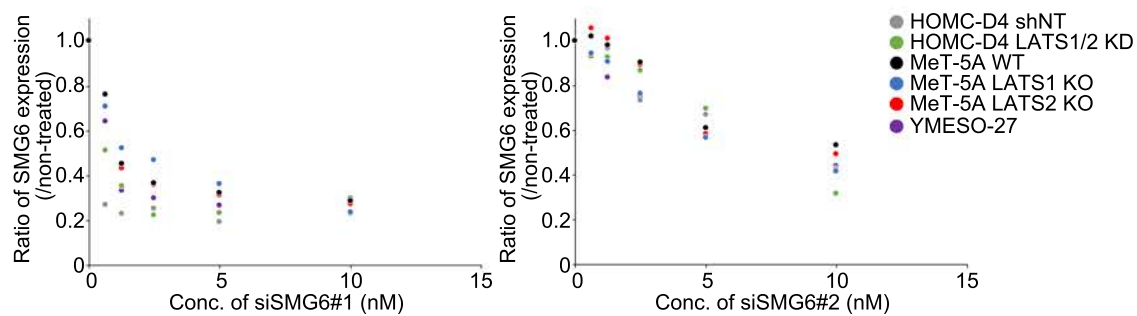**B**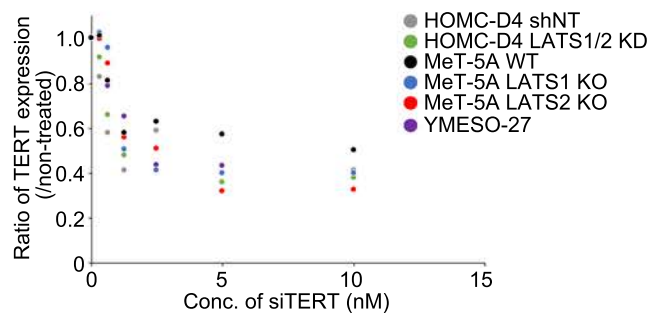**C**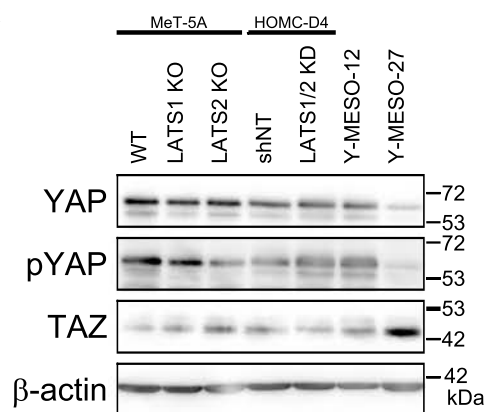

Figure S1 Suzuki et al.

Supplement: Supplementary file 2 — Supplemental Figure 1 [file 41420_2022_1232_MOESM2_ESM.pdf]

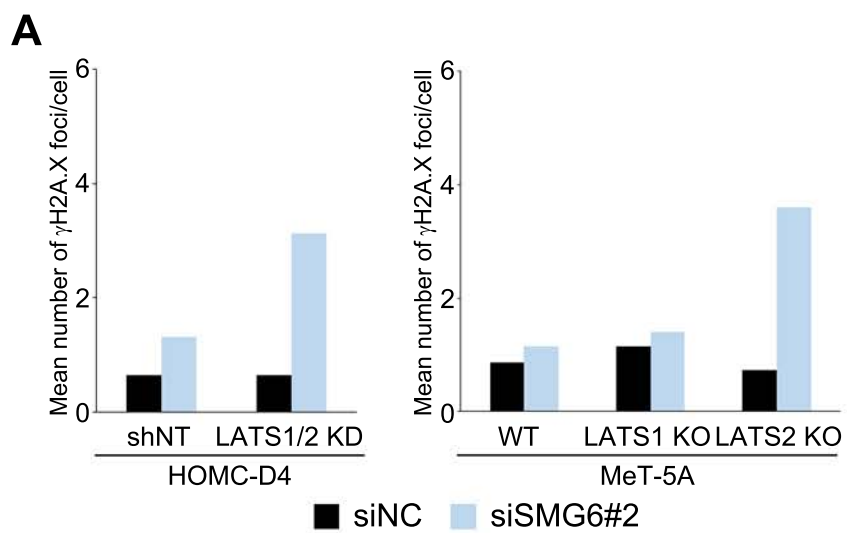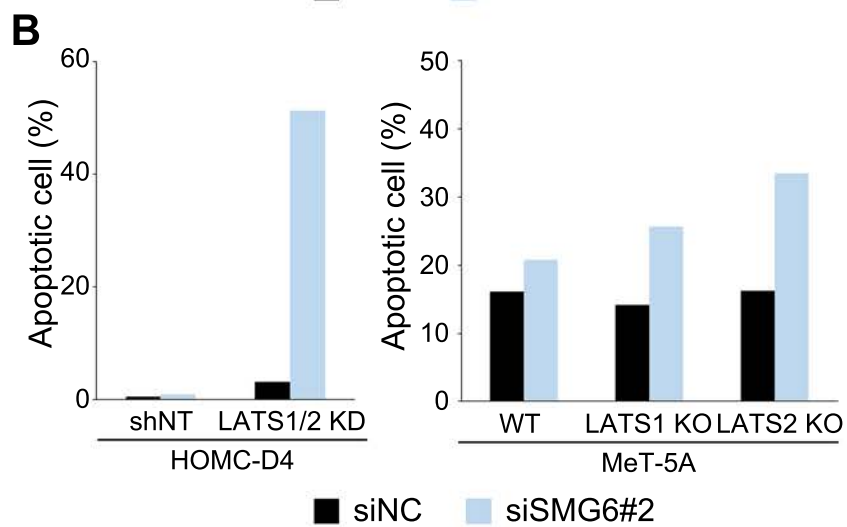

Figure S2 Suzuki et al.

Supplement: Supplementary file 3 — Supplemental Figure 2 [file 41420_2022_1232_MOESM3_ESM.pdf]

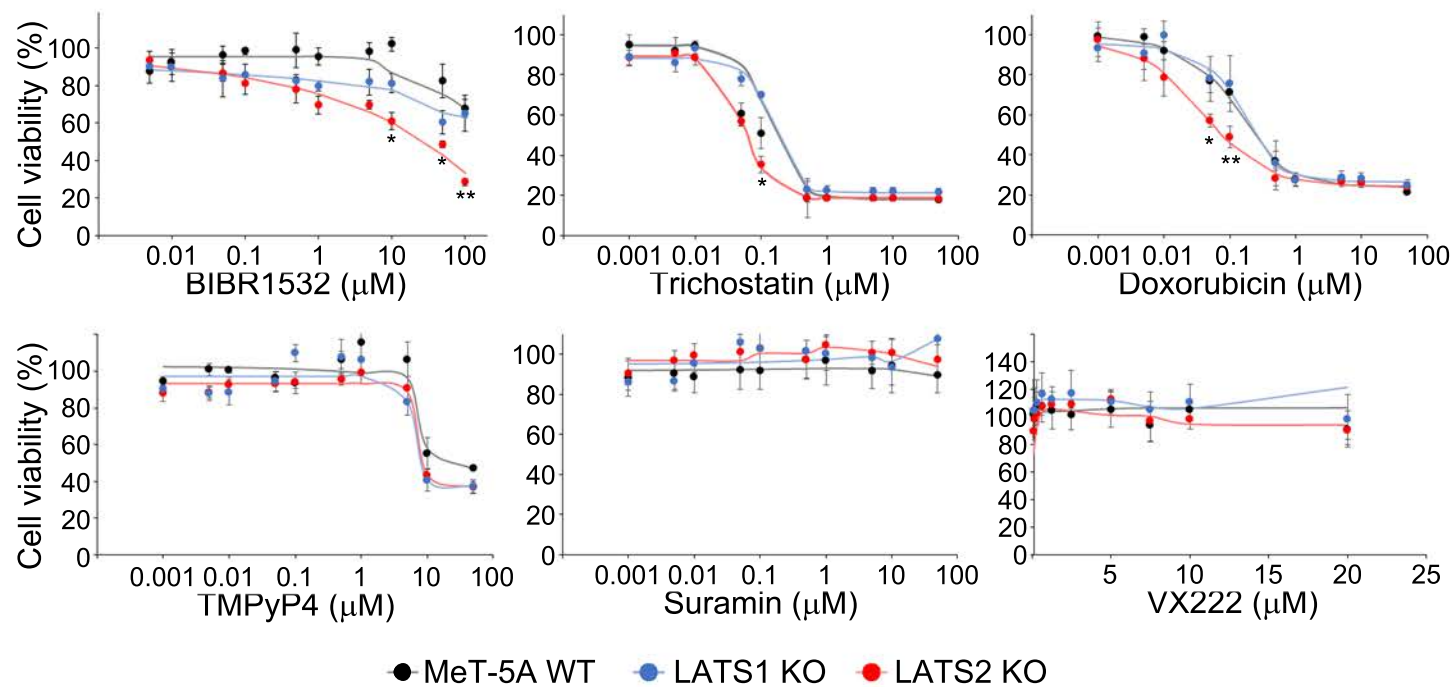

Figure S3 Suzuki et al.

Supplement: Supplementary file 4 — Supplemental Figure 3 [file 41420_2022_1232_MOESM4_ESM.pdf]

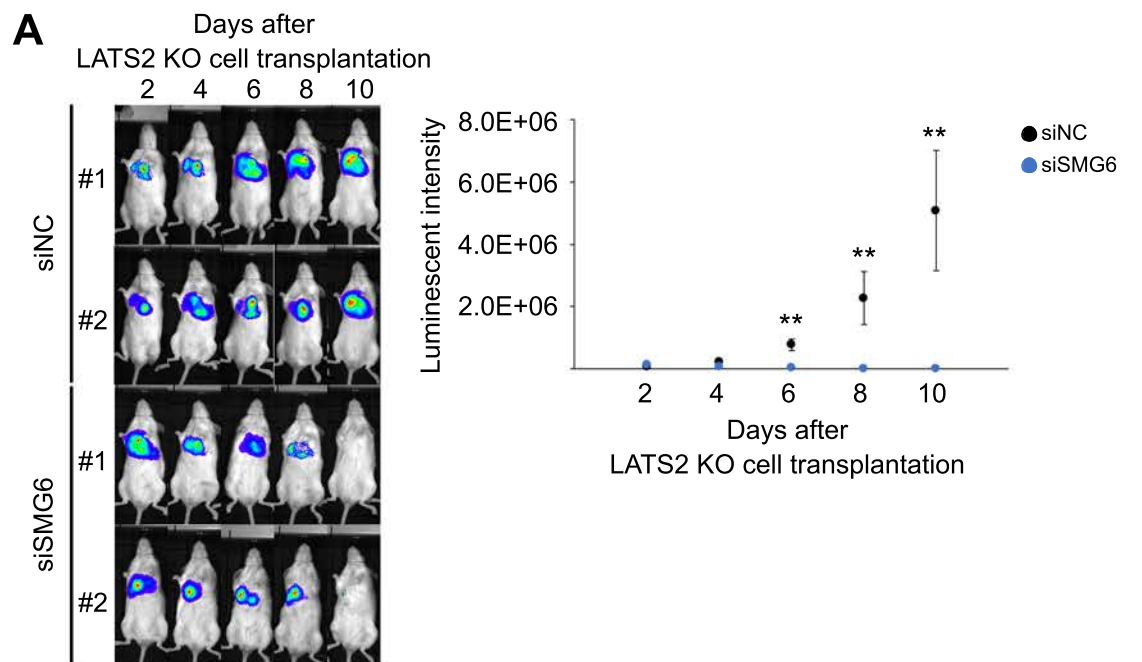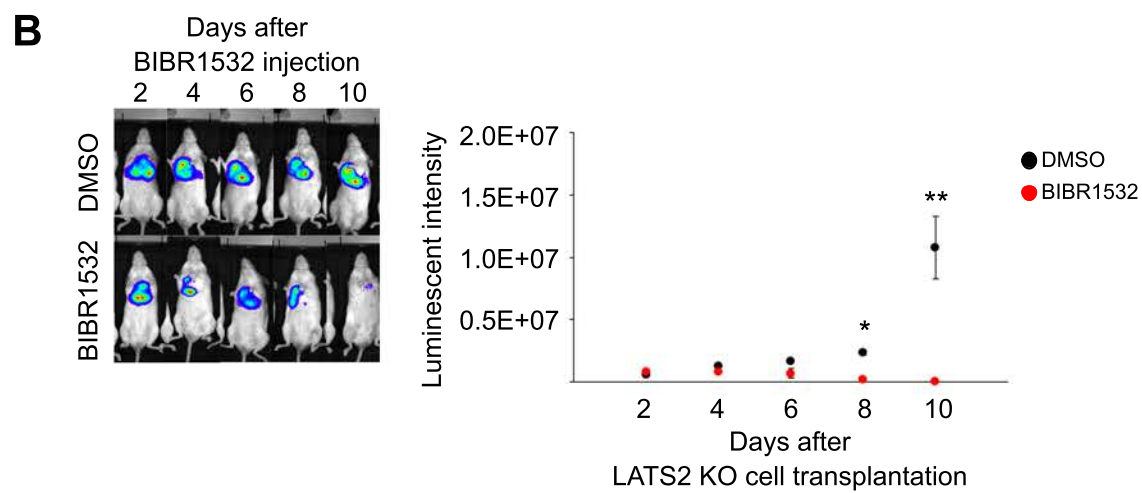

Figure S4 Suzuki et al.

Supplement: Supplementary file 5 — Supplemental Figure 4 [file 41420_2022_1232_MOESM5_ESM.pdf]

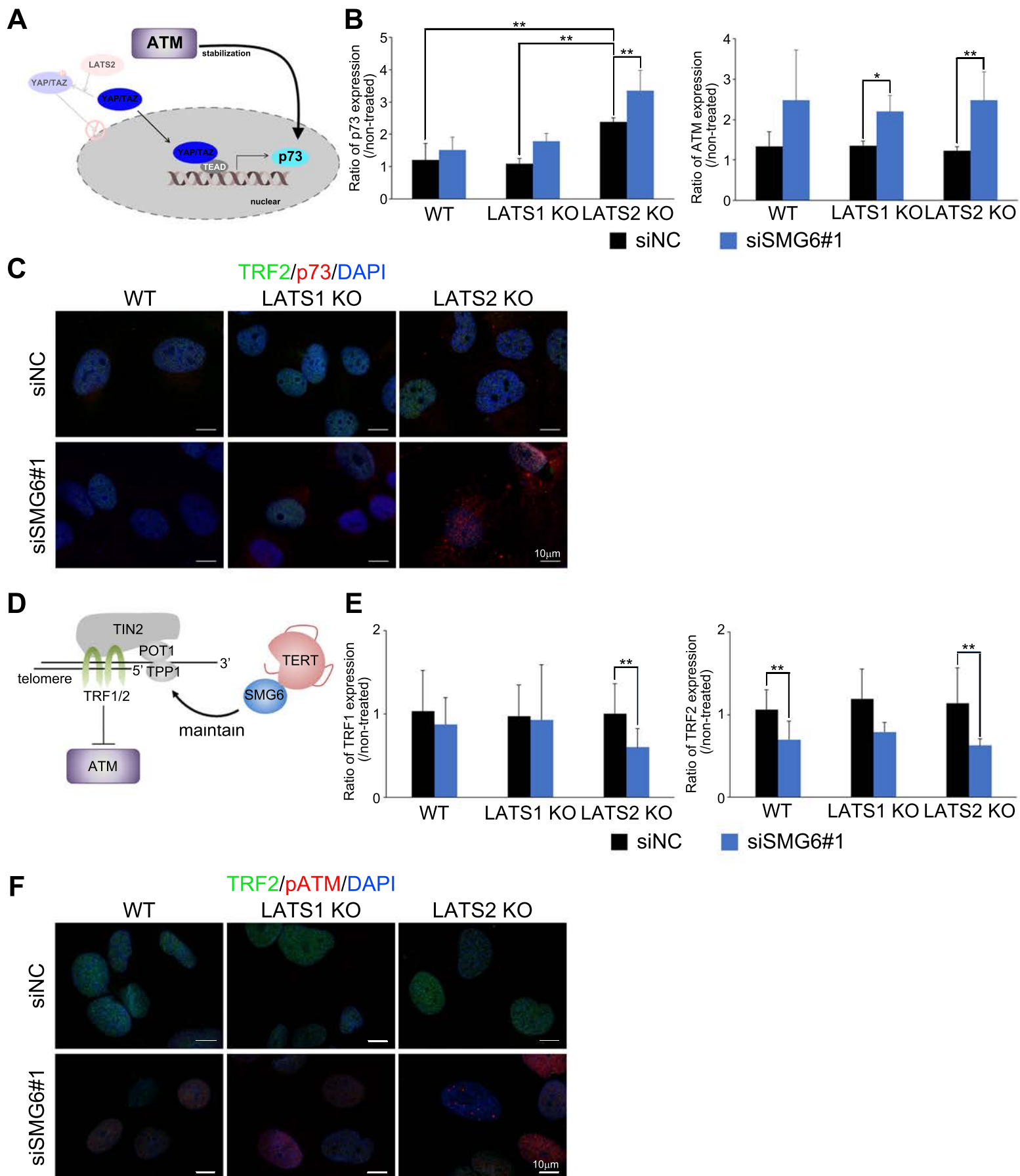

Figure S5 Suzuki et al.

Supplement: Supplementary file 6 — Supplemental Figure 5 [file 41420_2022_1232_MOESM6_ESM.pdf]

**A**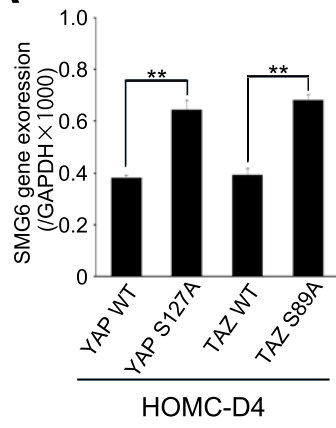**B**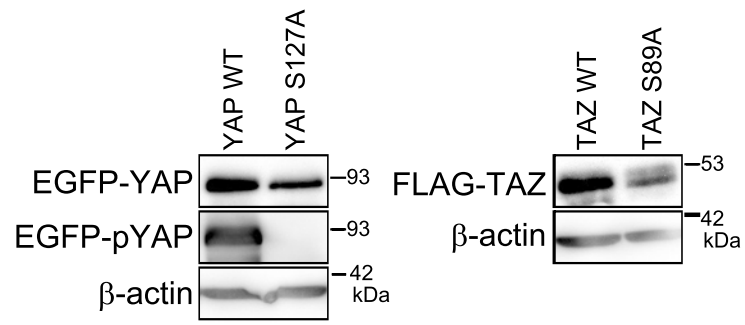

Figure S6 Suzuki et al.

Supplement: Supplementary file 7 — Supplemental Figure 6 [file 41420_2022_1232_MOESM7_ESM.pdf]

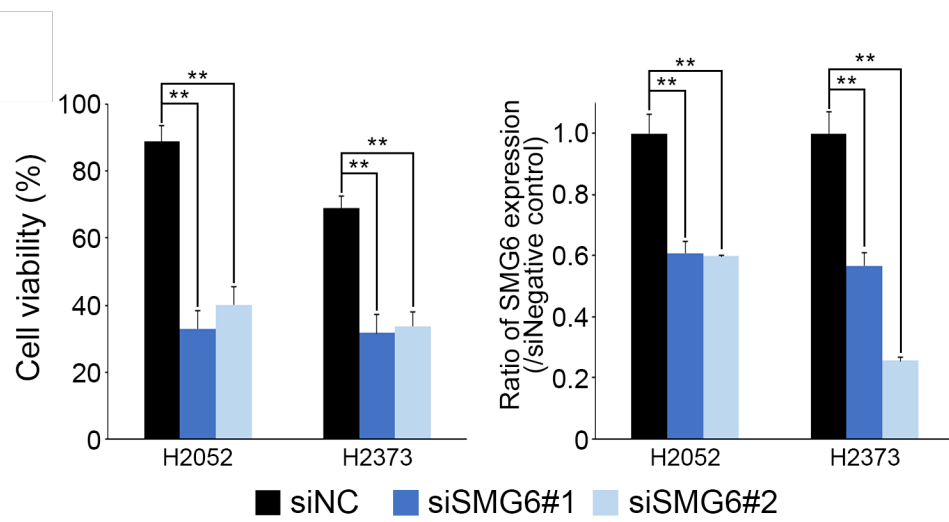

Figure S7 Suzuki et al.

Supplement: Supplementary file 8 — Supplemental Figure 7 [file 41420_2022_1232_MOESM8_ESM.pdf]

**A**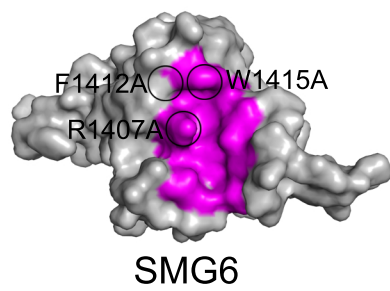**B**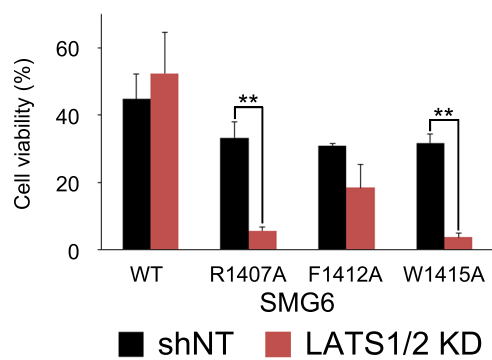

Figure S8 Suzuki et al.

Supplement: Supplementary file 9 — Supplemental Figure 8 [file 41420_2022_1232_MOESM9_ESM.pdf]

**A**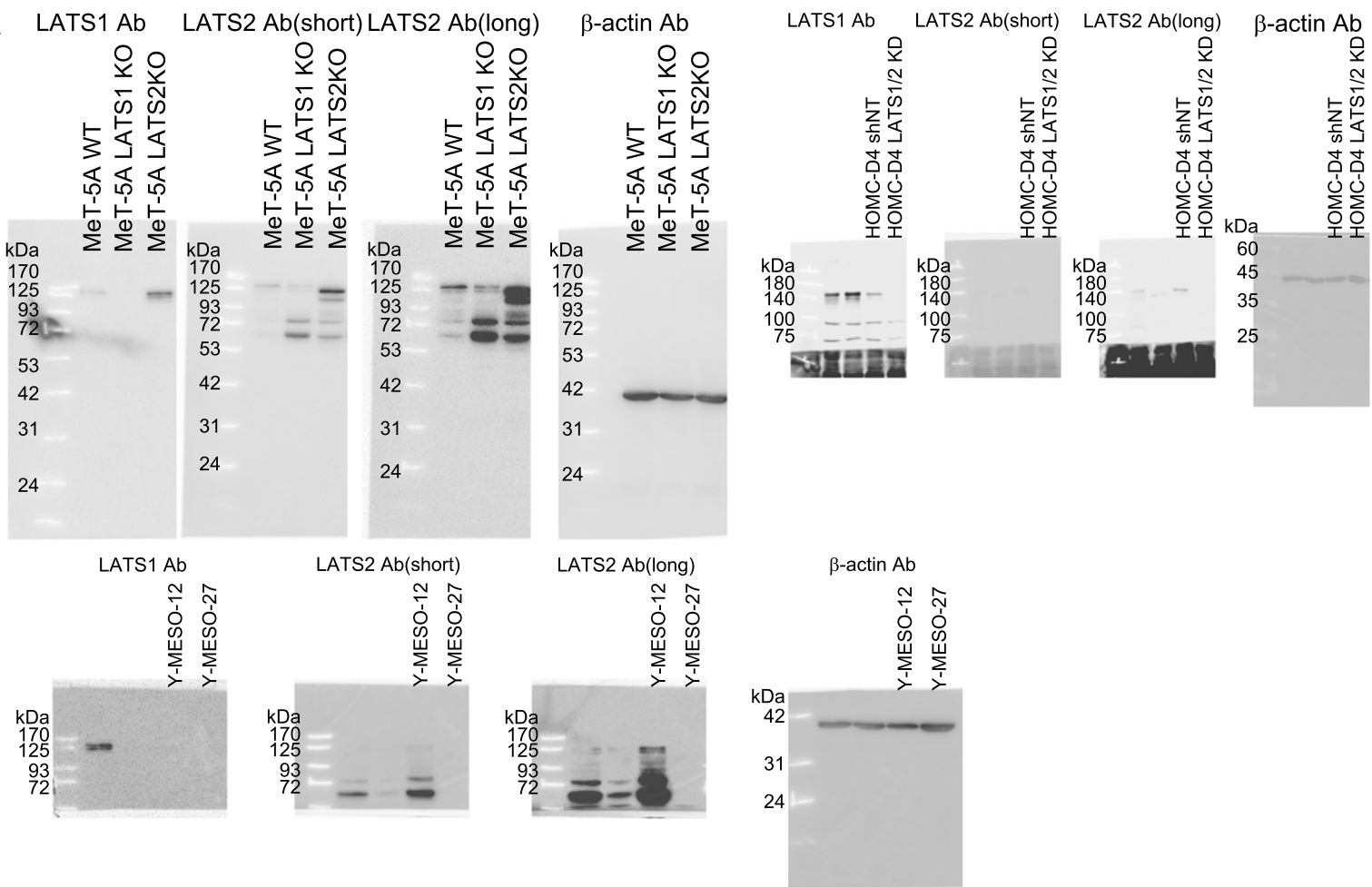**B**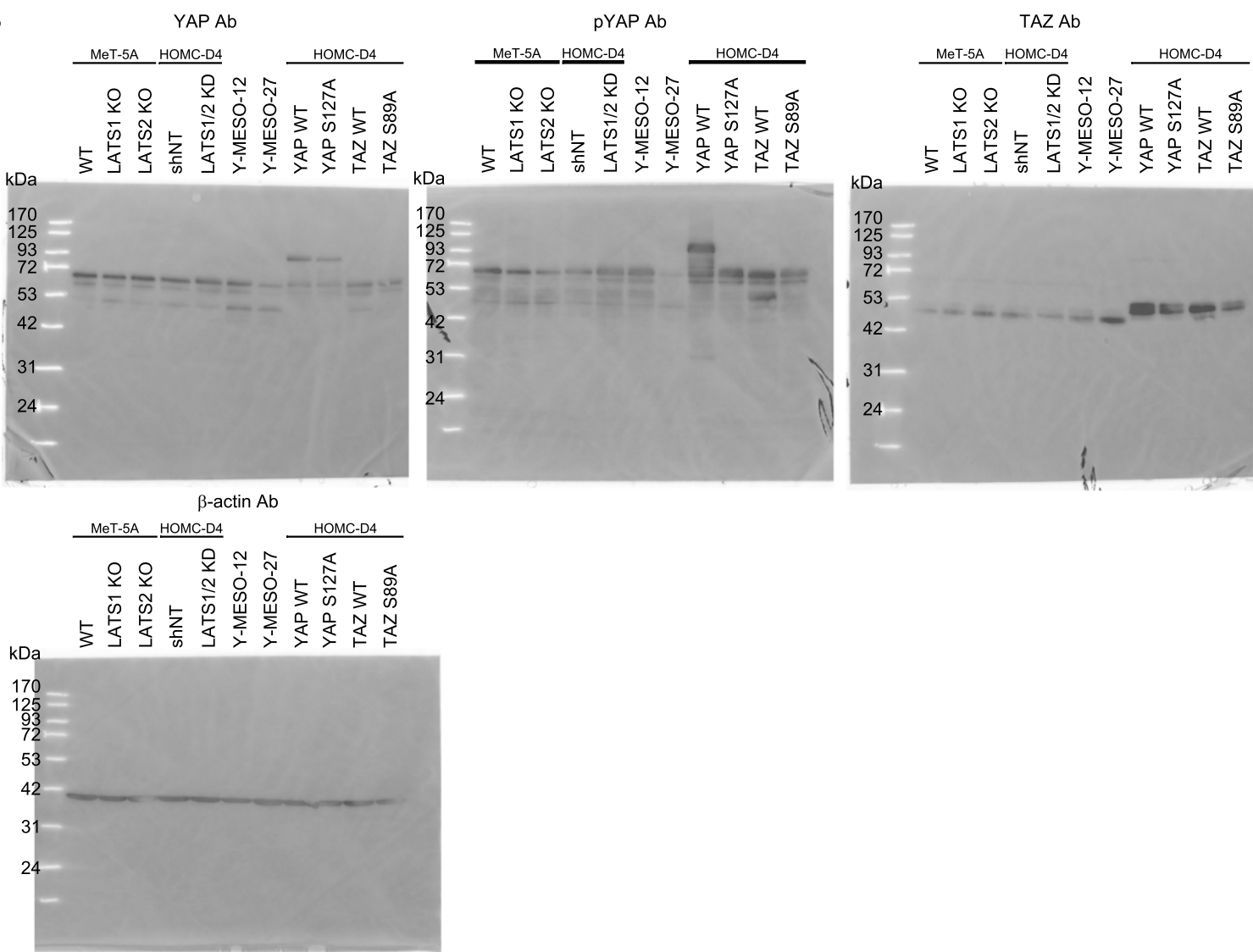

Figure S9 Suzuki et al.

Supplement: Supplementary file 10 — Supplemental Figure 9 [file 41420_2022_1232_MOESM10_ESM.pdf]
