# Supplementary material for: SMG6 regulates DNA damage and cell survival in Hippo pathway kinase LATS2-inactivated malignant mesothelioma
Source: Cell Death Discov. 2022 Nov 5;8:446. doi: 10.1038/s41420-022-01232-w (PMC9637146; doi:10.1038/s41420-022-01232-w)
Supplement: Supplementary file 11 — Confirmation the author names [file 41420_2022_1232_MOESM11_ESM.pdf]

差出人: **鈴木浩也** suzuki.kouya.iw@teikyo-u.ac.jp
件名: Re: Request to confirm the author names
日付: 2022年10月19日 18:39
宛先: murakamiyk@stf.teu.ac.jp

鈴木

Dear Prof.Yuko

I confirmed the manuscript.
There is no problem with this manuscript.

Sincerely

Koya Suzuki

2022年10月19日(水) 12:42 murakamiyk@stf.teu.ac.jp <murakamiyk@stf.teu.ac.jp>:

> Dear co-authors,
>
> Compared to the first draft, the number of authors has increased in the revised manuscript, and the order of author names has been changed due to changes in the level of contribution after experimenting with revisions. An email must be submitted to the Cell Death Discovery Editorial Office acknowledging that all co-authors have agreed to do so. There are no changes from the revised manuscript that you confirmed the other day, but please check it again and if there are no problems, please let me know.
>
> Sincerely,
>
> Yuko Murakami-Tonami
>
> *****

> MURAKAMI-TONAMI Yuko, MD & PhD
>
> Professor
> Cancer Molecular Genetics Lab,
> Graduate School of Bionics, Computer and Media Sciences,
> Tokyo University of Technology
>
> 1404- 1 Katakuramachi, Hachioji City,
> Tokyo 192-0983, JAPAN.
> Phone : +81-42-637-2756 (Office), 2460 (Lab)
> e-mail: murakamiyk@stf.teu.ac.jp
>
> Lab HP
> https://murakami-lab.bs.teu.ac.jp/

差出人: **丹下将希** g112101636@edu.teu.ac.jp
件名: Re: Request to confirm the author names
日付: 2022年10月19日 13:15
宛先: murakamiyk@stf.teu.ac.jp

丹

Dear Prof. Yuko Murakami-Tonami

My name is Masaki Tange, Tokyo University of Technology.
No problem about the author list.
Sincerely,
Masaki Tange

＊＊＊
TANGE MASAKI
Cancer Molecular Genetics Lab,
Graduate School of Bionics, Computer and Media Sciences, Tokyo University of Technology
E-mail g112101636@edu.teu.ac.jp
＊＊＊

2022年10月19日(水) 12:42 murakamiyk@stf.teu.ac.jp <murakamiyk@stf.teu.ac.jp>:

> Dear co-authors,
>
> Compared to the first draft, the number of authors has increased in the revised manuscript, and the order of author names has been changed due to changes in the level of contribution after experimenting with revisions. An email must be submitted to the Cell Death Discovery Editorial Office acknowledging that all co-authors have agreed to do so. There are no changes from the revised manuscript that you confirmed the other day, but please check it again and if there are no problems, please let me know.
>
> Sincerely,
>
> Yuko Murakami-Tonami
>
> *****
> MURAKAMI-TONAMI Yuko, MD & PhD
>
> Professor
> Cancer Molecular Genetics Lab,
> Graduate School of Bionics, Computer and Media Sciences,
> Tokyo University of Technology
>
> 1404- 1 Katakuramachi, Hachioji City,
> Tokyo 192-0983, JAPAN.
> Phone : +81-42-637-2756 (Office), 2460 (Lab)
> e-mail: murakamiyk@stf.teu.ac.jp
>
> Lab HP
> https://murakami-lab.bs.teu.ac.jp/

差出人: **山岸良多** yamagishi.ryota@omu.ac.jp
件名: Re: Request to confirm the author names
日付: 2022年10月19日 13:54
宛先: murakamiyk@stf.teu.ac.jp

山岸

Dear Dr. Murakami

Thank you for your email.
No problem for me.

Sincerely,
Ryota Yamagishi

****
Assistant Professor
Department of Pathophysiology
Osaka Metropolitan University Graduate School of Medicine
1-4-3 Asahimachi, Abeno-ku, Osaka, 545-8585, JAPAN
PHONE:+81-6-6645-3711
e-mail: yamagishi.ryota@omu.ac.jp
****

> 2022/10/19 12:42、murakamiyk@stf.teu.ac.jpのメール:
>
> Dear co-authors,
>
> Compared to the first draft, the number of authors has increased in the revised manuscript, and the order of author names has been changed due to changes in the level of contribution after experimenting with revisions. An email must be submitted to the Cell Death Discovery Editorial Office acknowledging that all co-authors have agreed to do so. There are no changes from the revised manuscript that you confirmed the other day, but please check it again and if there are no problems, please let me know.
>
> Sincerely,
>
> Yuko Murakami-Tonami
>
> *****
> MURAKAMI-TONAMI Yuko, MD & PhD
>
> Professor
> Cancer Molecular Genetics Lab,
> Graduate School of Bionics, Computer and Media Sciences,
> Tokyo University of Technology
>
> 1404- 1 Katakuramachi, Hachioji City,
> Tokyo 192-0983, JAPAN.
> Phone : +81-42-637-2756 (Office), 2460 (Lab)
> e-mail: murakamiyk@stf.teu.ac.jp
>
> Lab HP
> https://murakami-lab.bs.teu.ac.jp/

差出人: hiroyuki.hanada@riken.jp
件名: Re: Request to confirm the author names
日付: 2022年10月19日 13:22
宛先: murakamiyk@stf.teu.ac.jp

Dear Dr. Murakami,

This is Hiroyuki Hanada at RIKEN. Thank you for preparing for the submission.
I confirmed that the order of the authors, my name and my affiliation in the manuscript are fine for me.

From: murakamiyk@stf.teu.ac.jp <murakamiyk@stf.teu.ac.jp>
Sent: Wednesday, October 19, 2022 12:42
To: koya-suzuki@juntendo.ac.jp; 鈴木浩也; g112101636@edu.teu.ac.jp; 山岸良多; Hiroyuki Hanada; Satomi Mukai; Tatsuhiro Sato; 紅 朋浩; kkadoma@med.nagoya-u.ac.jp; phmaeda; 三井田孝; Ichiro Takeuchi; Ichiro Takeuchi; hmura@bio.chuo-u.ac.jp; Yoshitaka Sekido; tanaka@interprotein.com; Hirotsugu KOMATSU
Subject: Request to confirm the author names

Dear co-authors,

Compared to the first draft, the number of authors has increased in the revised manuscript, and the order of author names has been changed due to changes in the level of contribution after experimenting with revisions. An email must be submitted to the Cell Death Discovery Editorial Office acknowledging that all co-authors have agreed to do so. There are no changes from the revised manuscript that you confirmed the other day, but please check it again and if there are no problems, please let me know.
Sincerely,
Yuko Murakami-Tonami

*****
MURAKAMI-TONAMI Yuko, MD & PhD

Professor
Cancer Molecular Genetics Lab,
Graduate School of Bionics, Computer and Media Sciences,
Tokyo University of Technology

1404- 1 Katakuramachi, Hachioji City,
Tokyo 192-0983, JAPAN.
Phone : +81-42-637-2756 (Office), 2460 (Lab)
e-mail: murakamiyk@stf.teu.ac.jp<mailto:murakamiyk@stf.teu.ac.jp>

Lab HP
https://murakami-lab.bs.teu.ac.jp/

差出人: **Satomi Mukai** smukai@aichi-cc.jp
件名: Re: Request to confirm the author names
日付: 2022年10月19日 13:19
宛先: murakamiyk@stf.teu.ac.jp

MS

Prof. Murakami,

Thank you for contacting me again.
I agree to the changes.

Sincerely,
Satomi Mukai

--
****
Satomi Mukai
Div. Cancer Biol., Aichi Cancer Center Resarch Institute
1-1, Kanokoden, Chikusa-ku, Nagoya City, Aichi 464-8681, JAPAN
TEL: 052-764-2993
FAX: 052-763-5233
E-mail: smukai@aichi-cc.jp
****

On 2022/10/19 12:42, murakamiyk@stf.teu.ac.jp wrote:

> Dear co-authors,
>
> Compared to the first draft, the number of authors has increased in the revised manuscript, and the order of author names has been changed due to changes in the level of contribution after experimenting with revisions. An email must be submitted to the Cell Death Discovery Editorial Office acknowledging that all co-authors have agreed to do so. There are no changes from the revised manuscript that you confirmed the other day, but please check it again and if there are no problems, please let me know.
>
> Sincerely,
>
> Yuko Murakami-Tonami
>
> *****

MURAKAMI-TONAMI Yuko, MD & PhD

Professor
Cancer Molecular Genetics Lab,
Graduate School of Bionics, Computer and Media Sciences,
Tokyo University of Technology

1404- 1 Katakuramachi, Hachioji City,
Tokyo 192-0983, JAPAN.
Phone : +81-42-637-2756 (Office), 2460 (Lab)
e-mail: murakamiyk@stf.teu.ac.jp

Lab HP
https://murakami-lab.bs.teu.ac.jp/

--
****
向井　智美
愛知県がんセンター研究所
分子腫瘍学分野
〒464-8681
名古屋市千種区鹿子殿1番1号

[illegible]
TEL: 052-764-2993
FAX: 052-763-5233
E-mail: smukai@aichi-cc.jp
****

差出人: **Tatsuhiro Sato** satot@aichi-cc.jp
件名: Re: Request to confirm the author names
日付: 2022年10月19日 13:21
宛先: murakamiyk@stf.teu.ac.jp

ST

Dear Dr. Murakami-Tonami,

Thank you for your e-mail. I have no objection to my order in the list of authors in the manuscript.

Best regards.

Tatsuhiro Sato

Aichi Cancer Center Research Institute
Division of Cancer Biology
1-1 Kanokoden, Chikusa-ku, Nagoya, Aichi 90064, Japan
Tel: +81-52-762-6111
Email: satot@aichi-cc.jp

2022年10月19日(水) 12:42 murakamiyk@stf.teu.ac.jp <murakamiyk@stf.teu.ac.jp>:

> Dear co-authors,
>
> Compared to the first draft, the number of authors has increased in the revised manuscript, and the order of author names has been changed due to changes in the level of contribution after experimenting with revisions. An email must be submitted to the Cell Death Discovery Editorial Office acknowledging that all co-authors have agreed to do so. There are no changes from the revised manuscript that you confirmed the other day, but please check it again and if there are no problems, please let me know.
>
> Sincerely,
>
> Yuko Murakami-Tonami
>
> *****

> MURAKAMI-TONAMI Yuko, MD & PhD
>
> Professor
> Cancer Molecular Genetics Lab,
> Graduate School of Bionics, Computer and Media Sciences,
> Tokyo University of Technology
>
> 1404- 1 Katakuramachi, Hachioji City,
> Tokyo 192-0983, JAPAN.
> Phone : +81-42-637-2756 (Office), 2460 (Lab)
> e-mail: murakamiyk@stf.teu.ac.jp
>
> Lab HP
> https://murakami-lab.bs.teu.ac.jp/

差出人: **Takeshi TANAKA** tanaka@interprotein.com
件名: Re: Request to confirm the author names
日付: 2022年10月19日 14:51
宛先: murakamiyk@stf.teu.ac.jp
CC: kkadoma@med.nagoya-u.ac.jp、 koya-suzuki@juntendo.ac.jp、 鈴木浩也 suzuki.kouya.iw@teikyo-u.ac.jp、 g112101636@edu.teu.ac.jp、 山岸良多 yamagishi.ryota@omu.ac.jp、 Hiroyuki Hanada hiroyuki.hanada@riken.jp、 Satomi Mukai smukai@aichi-cc.jp、 Tatsuhiro Sato satot@aichi-cc.jp、 紅 朋浩 akashi@med.nagoya-u.ac.jp、 phmaeda phmaeda@kinjo-u.ac.jp、 三井田孝 tmiida@juntendo.ac.jp、 Ichiro Takeuchi takeuchi.ichiro@nitech.ac.jp、 Ichiro Takeuchi itakeuchi73@gmail.com、 hmura@bio.chuo-u.ac.jp、 Yoshitaka Sekido ysekido@aichi-cc.jp

---

Dear Dr. Yuko,

I have confirmed it. There is no problem.

Takeshi

Takeshi TANAKA Ph. D.
Director for Drug Design Department
Interprotein Corporation
www.interprotein.com
SIC-2-2205, 5-4-30, Nishihashimoto, Midori-ku,
Sagamihara, Kanagawa, 252-0131, Japan
tel/fax: +81-42-770-9477
e-mail: tanaka@interprotein.com

> 2022/10/19 PM1:06、<kkadoma@med.nagoya-u.ac.jp> <kkadoma@med.nagoya-u.ac.jp>のメール:
>
> Dear Yuko,
>
> No problem for me.
>
> Kenji
>
> Kenji Kadomatsu MD, PhD
> Professor
> Department of Biochemistry
> Nagoya University Graduate School of Medicine
> 65 Tsurumai-cho, Showa-ku
> Nagoya 466-8550
> Japan
> Tel: +81-52-744-2060
> Fax: +81-52-744-2065
>
> From: murakamiyk@stf.teu.ac.jp <murakamiyk@stf.teu.ac.jp>
> Sent: Wednesday, October 19, 2022 12:42 PM
> To: koya-suzuki@juntendo.ac.jp; 鈴木浩也 <suzuki.kouya.iw@teikyo-u.ac.jp>; g112101636@edu.teu.ac.jp; 山岸良多 <yamagishi.ryota@omu.ac.jp>; Hiroyuki Hanada <hiroyuki.hanada@riken.jp>; Satomi Mukai <smukai@aichi-cc.jp>; Tatsuhiro Sato <satot@aichi-cc.jp>; 紅 朋浩 <akashi@med.nagoya-u.ac.jp>; kkadoma@med.nagoya-u.ac.jp; phmaeda <phmaeda@kinjo-u.ac.jp>; 三井田孝 <tmiida@juntendo.ac.jp>; Ichiro Takeuchi <takeuchi.ichiro@nitech.ac.jp>; Ichiro Takeuchi <itakeuchi73@gmail.com>; hmura@bio.chuo-u.ac.jp; Yoshitaka Sekido <ysekido@aichi-cc.jp>; tanaka@interprotein.com; Hirotsugu KOMATSU <komatsu@interprotein.com>
> Subject: Request to confirm the author names
>
> Dear co-authors,
>
> Compared to the first draft, the number of authors has increased in the revised manuscript, and the order of author names has been changed due to changes in the level of contribution after experimenting with revisions. An email must be submitted to the Cell Death Discovery Editorial Office acknowledging that all co-authors have agreed to do so. There are no changes from the revised manuscript that you confirmed the other day, but please check it again and if there are no problems, please let me know.
> Sincerely,
> Yuko Murakami-Tonami
>
> *****
> MURAKAMI-TONAMI Yuko, MD & PhD
>
> Professor
> Cancer Molecular Genetics Lab,
> Graduate School of Bionics, Computer and Media Sciences,
> Tokyo University of Technology
>
> 1404- 1 Katakuramachi, Hachioji City

[illegible]
Tokyo 192-0983, JAPAN.
Phone : +81-42-637-2756 (Office), 2460 (Lab)
e-mail: murakamiyk@stf.teu.ac.jp

Lab HP
https://murakami-lab.bs.teu.ac.jp/

差出人: **紅 朋浩** akashi@med.nagoya-u.ac.jp
件名: Re: Request to confirm the author names
日付: 2022年10月19日 15:11
宛先: murakamiyk@stf.teu.ac.jp

紅

Dear Dr. Yuko Murakami-Tonami,

I agree to the changes without any problems. Thanks!

Sincerely,

Tomohiro Akashi, Ph.D.
Division of Systems Biology
Department of Integrative Cellular Informatics
Center for Neurological Diseases and Cancer
Nagoya University Graduate School of Medicine

> 2022/10/19 12:42、murakamiyk@stf.teu.ac.jpのメール:
>
> Dear co-authors,
>
> Compared to the first draft, the number of authors has increased in the revised manuscript, and the order of author names has been changed due to changes in the level of contribution after experimenting with revisions. An email must be submitted to the Cell Death Discovery Editorial Office acknowledging that all co-authors have agreed to do so. There are no changes from the revised manuscript that you confirmed the other day, but please check it again and if there are no problems, please let me know.
>
> Sincerely,
>
> Yuko Murakami-Tonami
>
> *****

MURAKAMI-TONAMI Yuko, MD & PhD

Professor
Cancer Molecular Genetics Lab,
Graduate School of Bionics, Computer and Media Sciences,
Tokyo University of Technology

1404- 1 Katakuramachi, Hachioji City,
Tokyo 192-0983, JAPAN.
Phone : +81-42-637-2756 (Office), 2460 (Lab)
e-mail: murakamiyk@stf.teu.ac.jp

Lab HP
https://murakami-lab.bs.teu.ac.jp/

差出人: kkadoma@med.nagoya-u.ac.jp
件名: RE: Request to confirm the author names
日付: 2022年10月19日 13:06
宛先: murakamiyk@stf.teu.ac.jp、 koya-suzuki@juntendo.ac.jp、 鈴木浩也 suzuki.kouya.iw@teikyo-u.ac.jp、 g112101636@edu.teu.ac.jp、 山岸良多 yamagishi.ryota@omu.ac.jp、 Hiroyuki Hanada hiroyuki.hanada@riken.jp、 Satomi Mukai smukai@aichi-cc.jp、 Tatsuhiro Sato satot@aichi-cc.jp、 紅 朋浩 akashi@med.nagoya-u.ac.jp、 phmaeda phmaeda@kinjo-u.ac.jp、 三井田孝 tmiida@juntendo.ac.jp、 Ichiro Takeuchi takeuchi.ichiro@nitech.ac.jp、 Ichiro Takeuchi itakeuchi73@gmail.com、 hmura@bio.chuo-u.ac.jp、 Yoshitaka Sekido ysekido@aichi-cc.jp、 tanaka@interprotein.com、 Hirotsugu KOMATSU komatsu@interprotein.com

---

Dear Yuko,

No problem for me.

Kenji

Kenji Kadomatsu MD, PhD
Professor
Department of Biochemistry
Nagoya University Graduate School of Medicine
65 Tsurumai-cho, Showa-ku
Nagoya 466-8550
Japan
Tel: +81-52-744-2060
Fax: +81-52-744-2065

From: murakamiyk@stf.teu.ac.jp <murakamiyk@stf.teu.ac.jp>
Sent: Wednesday, October 19, 2022 12:42 PM
To: koya-suzuki@juntendo.ac.jp; 鈴木浩也 <suzuki.kouya.iw@teikyo-u.ac.jp>; g112101636@edu.teu.ac.jp; 山岸良多 <yamagishi.ryota@omu.ac.jp>; Hiroyuki Hanada <hiroyuki.hanada@riken.jp>; Satomi Mukai <smukai@aichi-cc.jp>; Tatsuhiro Sato <satot@aichi-cc.jp>; 紅 朋浩 <akashi@med.nagoya-u.ac.jp>; kkadoma@med.nagoya-u.ac.jp; phmaeda <phmaeda@kinjo-u.ac.jp>; 三井田孝 <tmiida@juntendo.ac.jp>; Ichiro Takeuchi <takeuchi.ichiro@nitech.ac.jp>; Ichiro Takeuchi <itakeuchi73@gmail.com>; hmura@bio.chuo-u.ac.jp; Yoshitaka Sekido <ysekido@aichi-cc.jp>; tanaka@interprotein.com; Hirotsugu KOMATSU <komatsu@interprotein.com>
Subject: Request to confirm the author names

Dear co-authors,

Compared to the first draft, the number of authors has increased in the revised manuscript, and the order of author names has been changed due to changes in the level of contribution after experimenting with revisions. An email must be submitted to the Cell Death Discovery Editorial Office acknowledging that all co-authors have agreed to do so. There are no changes from the revised manuscript that you confirmed the other day, but please check it again and if there are no problems, please let me know.
Sincerely,
Yuko Murakami−Tonami

*****
MURAKAMI-TONAMI Yuko, MD & PhD

Professor

[illegible]
Cancer Molecular Genetics Lab,
Graduate School of Bionics, Computer and Media Sciences,
Tokyo University of Technology

1404- 1 Katakuramachi, Hachioji City,
Tokyo 192-0983, JAPAN.
Phone : +81-42-637-2756 (Office), 2460 (Lab)
e-mail: murakamiyk@stf.teu.ac.jp

Lab HP
https://murakami-lab.bs.teu.ac.jp/

差出人: **phmaeda** phmaeda@kinjo-u.ac.jp
件名: Re: Request to confirm the author names
日付: 2022年10月19日 18:19
宛先: murakamiyk@stf.teu.ac.jp

P

Dear Dr. Yuko,

I have confirmed it. There is no problem.

Tohru Maeda

2022年10月19日(水) 12:42 murakamiyk@stf.teu.ac.jp <murakamiyk@stf.teu.ac.jp>:

> Dear co-authors,
>
> Compared to the first draft, the number of authors has increased in the revised manuscript, and the order of author names has been changed due to changes in the level of contribution after experimenting with revisions. An email must be submitted to the Cell Death Discovery Editorial Office acknowledging that all co-authors have agreed to do so. There are no changes from the revised manuscript that you confirmed the other day, but please check it again and if there are no problems, please let me know.
>
> Sincerely,
>
> Yuko Murakami-Tonami
>
> *****

> MURAKAMI-TONAMI Yuko, MD & PhD
>
> Professor
> Cancer Molecular Genetics Lab,
> Graduate School of Bionics, Computer and Media Sciences,
> Tokyo University of Technology
>
> 1404- 1 Katakuramachi, Hachioji City,
> Tokyo 192-0983, JAPAN.
> Phone : +81-42-637-2756 (Office), 2460 (Lab)
> e-mail: murakamiyk@stf.teu.ac.jp
>
> Lab HP
> https://murakami-lab.bs.teu.ac.jp/

--
前田　徹

****
前田　徹

金城学院大学薬学部
　実務医療系分野　教授
〒463-8521　名古屋市守山区大森2-1723
TEL　052-798-0180（内線762）
E-mail phmaeda@kinjo-u.ac.jp
****************************強く、優しく。**

差出人: 三井田孝 tmiida@juntendo.ac.jp
件名: Re: Request to confirm the author names
日付: 2022年10月19日 22:26
宛先: murakamiyk@stf.teu.ac.jp
CC: koya-suzuki@juntendo.ac.jp、 鈴木浩也 suzuki.kouya.iw@teikyo-u.ac.jp、 g112101636@edu.teu.ac.jp、 山岸良多 yamagishi.ryota@omu.ac.jp、 Hiroyuki Hanada hiroyuki.hanada@riken.jp、 Satomi Mukai smukai@aichi-cc.jp、 Tatsuhiro Sato satot@aichi-cc.jp、 紅 朋浩 akashi@med.nagoya-u.ac.jp、 kkadoma@med.nagoya-u.ac.jp、 phmaeda phmaeda@kinjo-u.ac.jp、 Ichiro Takeuchi takeuchi.ichiro@nitech.ac.jp、 Ichiro Takeuchi itakeuchi73@gmail.com、 hmura@bio.chuo-u.ac.jp、 Yoshitaka Sekido ysekido@aichi-cc.jp、 tanaka@interprotein.com、 Hirotsugu KOMATSU komatsu@interprotein.com

---

Dear Prof. Yuko Murakami-Tonami,

Thank you for your email. Congratulations on the interim acceptance of our paper.

,
I have already agreed that the number of co-authors increased in the latest version, and that the order of the authors were changed from that of the original version. I have no objection to these changes.

I am looking forward to hearing from you soon,

Best wishes,

Takashi
****
Takashi Miida, M. D., Ph. D.

Department of Clinical Laboratory Medicine,
Juntendo University　Graduate School of Medicine
Tokyo 113-8421
Japan

TEL: +81-3-5802-1104
FAX：+81-3-5684-1609
E-mail: tmiida@juntendo.ac.jp
****

2022年10月19日(水) 12:42 murakamiyk@stf.teu.ac.jp <murakamiyk@stf.teu.ac.jp>:

> Dear co-authors,
>
> Compared to the first draft, the number of authors has increased in the revised manuscript, and the order of author names has been changed due to changes in the level of contribution after experimenting with revisions. An email must be submitted to the Cell Death Discovery Editorial Office acknowledging that all co-authors have agreed to do so. There are no changes from the revised manuscript that you confirmed the other day, but please check it again and if there are no problems, please let me know.
>
> Sincerely,
>
> Yuko Murakami-Tonami
>
> *****
> MURAKAMI-TONAMI Yuko, MD & PhD
>
> Professor
> Cancer Molecular Genetics Lab,
> Graduate School of Bionics, Computer and Media Sciences,
> Tokyo University of Technology
>
> 1404- 1 Katakuramachi, Hachioji City,
> Tokyo 192-0983, JAPAN.
> Phone : +81-42-637-2756 (Office), 2460 (Lab)
> e-mail: murakamiyk@stf.teu.ac.jp
>
> Lab HP
> https://murakami-lab.bs.teu.ac.jp/

差出人: **Ichiro Takeuchi** itakeuchi73@gmail.com
件名: Re: Request to confirm the author names
日付: 2022年10月19日 14:54
宛先: murakamiyk@stf.teu.ac.jp

TI

Dear Yuko

It is no problem for me. Thanks!

Ichiro Takeuchi

Ichiro TAKEUCHI, Professor, Nagoya University / Team Leader, RIKEN AIP
E-mail: ichiro.takeuchi@mae.nagoya-u.ac.jp / itakeuchi73@gmail.com
Tel: 052-789-2739

2022年10月19日(水) 12:42 murakamiyk@stf.teu.ac.jp <murakamiyk@stf.teu.ac.jp>:

> Dear co-authors,
>
> Compared to the first draft, the number of authors has increased in the revised manuscript, and the order of author names has been changed due to changes in the level of contribution after experimenting with revisions. An email must be submitted to the Cell Death Discovery Editorial Office acknowledging that all co-authors have agreed to do so. There are no changes from the revised manuscript that you confirmed the other day, but please check it again and if there are no problems, please let me know.
>
> Sincerely,
>
> Yuko Murakami-Tonami
>
> *****

MURAKAMI-TONAMI Yuko, MD & PhD

Professor
Cancer Molecular Genetics Lab,
Graduate School of Bionics, Computer and Media Sciences,
Tokyo University of Technology

1404- 1 Katakuramachi, Hachioji City,
Tokyo 192-0983, JAPAN.
Phone : +81-42-637-2756 (Office), 2460 (Lab)
e-mail: murakamiyk@stf.teu.ac.jp

Lab HP
https://murakami-lab.bs.teu.ac.jp/

差出人: **村上 浩士** hmura@bio.chuo-u.ac.jp
件名: Re: Request to confirm the author names
日付: 2022年10月19日 13:44
宛先: murakamiyk@stf.teu.ac.jp

Dear Yuko

Thank you for letting me know the status.
I checked it and found no problems.

Best wishes

Hiroshi Murakami
--
Hiroshi Murakami, PhD
Professor

Department of Biological Science
Faculty of Science and Engineering
Chuo University
1-13-27 Kasuga, Bunkyo-ku, Tokyo 112-8551, Japan
Tel: +81-3-3817-7200
Fax: +81-3-3817-7102
hmura@bio.chuo-u.ac.jp

> 2022/10/19 12:42、murakamiyk@stf.teu.ac.jpのメール:
>
> Dear co-authors,
>
> Compared to the first draft, the number of authors has increased in the revised manuscript, and the order of author names has been changed due to changes in the level of contribution after experimenting with revisions. An email must be submitted to the Cell Death Discovery Editorial Office acknowledging that all co-authors have agreed to do so. There are no changes from the revised manuscript that you confirmed the other day, but please check it again and if there are no problems, please let me know.
>
> Sincerely,
>
> Yuko Murakami-Tonami
>
> *****

> MURAKAMI-TONAMI Yuko, MD & PhD
>
> Professor
> Cancer Molecular Genetics Lab,
> Graduate School of Bionics, Computer and Media Sciences,
> Tokyo University of Technology
>
> 1404- 1 Katakuramachi, Hachioji City,
> Tokyo 192-0983, JAPAN.
> Phone : +81-42-637-2756 (Office), 2460 (Lab)
> e-mail: murakamiyk@stf.teu.ac.jp
>
> Lab HP
> https://murakami-lab.bs.teu.ac.jp/

差出人: **Yoshitaka Sekido** ysekido@aichi-cc.jp
件名: RE: Request to confirm the author names
日付: 2022年10月20日 8:42
宛先: murakamiyk@stf.teu.ac.jp、 koya-suzuki@juntendo.ac.jp、 鈴木浩也 suzuki.kouya.iw@teikyo-u.ac.jp、 g112101636@edu.teu.ac.jp、 山岸良多 yamagishi.ryota@omu.ac.jp、 Hiroyuki Hanada hiroyuki.hanada@riken.jp、 向井 智美 smukai@aichi-cc.jp、 Tatsuhiro Sato satot@aichi-cc.jp、 紅 朋浩 akashi@med.nagoya-u.ac.jp、 kkadoma@med.nagoya-u.ac.jp、 前田徹 phmaeda@kinjo-u.ac.jp、 三井田孝 tmiida@juntendo.ac.jp、 Ichiro Takeuchi takeuchi.ichiro@nitech.ac.jp、 Ichiro Takeuchi itakeuchi73@gmail.com、 hmura@bio.chuo-u.ac.jp、 tanaka@interprotein.com 、 Hirotsugu KOMATSU komatsu@interprotein.com

SY

Dear Yuko,

Thanks for your email.
I again confirmed and agreed with the authorship of the latest manuscript.

I hope everything goes well.

Best,

Yoshi

Yoshitaka Sekido M.D, Ph.D.

Vice Director, Chief
Division of Cancer Biology
Aichi Cancer Center Research Institute
Kanokoden 1-1, Chikusa-ku, Nagoya, Aichi
464-8681, JAPAN
+81-52-764-2983 (Direct)

From: murakamiyk@stf.teu.ac.jp <murakamiyk@stf.teu.ac.jp>
Sent: Wednesday, October 19, 2022 12:42 PM
To: koya-suzuki@juntendo.ac.jp; 鈴木浩也 <suzuki.kouya.iw@teikyo-u.ac.jp>; g112101636@edu.teu.ac.jp; 山岸良多 <yamagishi.ryota@omu.ac.jp>; Hiroyuki Hanada <hiroyuki.hanada@riken.jp>; 向井 智美 <smukai@aichi-cc.jp>; Tatsuhiro Sato <satot@aichi-cc.jp>; 紅 朋浩 <akashi@med.nagoya-u.ac.jp>; kkadoma@med.nagoya-u.ac.jp; 前田徹 <phmaeda@kinjo-u.ac.jp>; 三井田孝 <tmiida@juntendo.ac.jp>; Ichiro Takeuchi <takeuchi.ichiro@nitech.ac.jp>; Ichiro Takeuchi <itakeuchi73@gmail.com>; hmura@bio.chuo-u.ac.jp; Yoshitaka Sekido <ysekido@aichi-cc.jp>; tanaka@interprotein.com; Hirotsugu KOMATSU <komatsu@interprotein.com>
Subject: Request to confirm the author names

Dear co-authors,

Compared to the first draft, the number of authors has increased in the revised manuscript, and the order of author names has been changed due to changes in the level of contribution after experimenting with revisions. An email must be submitted to the Cell Death Discovery Editorial Office acknowledging that all co-authors have agreed to do so. There are no changes from the revised manuscript that you confirmed the other day, but please check it again and if there are no problems, please let me know.
Sincerely,
Yuko Murakami-Tonami

*****

MURAKAMI-TONAMI Yuko, MD & PhD

Professor
Cancer Molecular Genetics Lab,
Graduate School of Bionics, Computer and Media Sciences,
Tokyo University of Technology

1404- 1 Katakuramachi, Hachioji City,
Tokyo 192-0983, JAPAN.
Phone : +81-42-637-2756 (Office), 2460 (Lab)
e-mail: murakamiyk@stf.teu.ac.jp

Lab HP
https://murakami-lab.bs.teu.ac.jp/
